# Assessing Carbon Stock and Sequestration Capacity of *Phragmites australis* Using Integrated Destructive Sampling and Remote Sensing Approaches in Arid Environments

**DOI:** 10.3390/plants15213416

**Published:** 2026-11-03

**Authors:** Khaled Al-Jabri, Yaseen Al-Mulla, Mohammed Al Sulaimani, Hameed Sulaiman, Ahmed Tabook, Ahmed Al-Abri

**Affiliations:** 1 Department of Soils, Water and Agricultural Engineering, Sultan Qaboos University, Al-Khoud 123, Oman; 2 Remote Sensing and GIS Research Center, Sultan Qaboos University, Al-Khoud 123, Oman; 3 Petroleum Development Oman, Al-Qurum 100, Oman; mohammed.ms.sulaimani@pdo.co.om (M.A.S.); ahmed.s.tabook@pdo.co.om (A.T.); ahmed.am.abri@pdo.co.om (A.A.-A.); 4 Department of Biology, College of Science, Sultan Qaboos University, Al-Khoud 123, Oman; hameed@squ.edu.om

**Keywords:** *Phragmites australis*, aboveground biomass, carbon sequestration, destructive method, GIS, remote sensing

## Abstract

*Phragmites australis* (common reed) delivers key ecosystem services, including carbon sequestration, yet its carbon storage potential in hyper-arid environments remains insufficiently quantified. This study combines destructive sampling, laboratory carbon analysis, and satellite-derived vegetation indices to establish a rigorously validated baseline for aboveground biomass (AGB) and carbon stocks in a constructed wetland in Oman (n = 12 plots). Using leave-one-out cross-validation, this study tested whether biomass-prediction approaches commonly applied in wetland carbon research, namely height-based allometric equations and satellite vegetation indices, generalize beyond the data used to build them. The results show that the height-based equations achieve a near-perfect statistical fit by mathematical construction rather than genuine predictive validity, and that satellite vegetation indices require a larger, independent sample than used here to achieve validated accuracy; together, these findings establish a concrete, quantitative benchmark for sampling design and model validation in data-limited hyper-arid systems. The corrected destructive-sampling dataset places carbon stock at this site between 0.19 and 1.95 kg/m^2^, depending on the measurement method used. Together, these results support *P. australis*-dominated constructed wetlands as measurable, nature-based carbon reservoirs and chart a clear, evidence-based path toward reliable, remote sensing-based carbon monitoring across the Gulf Cooperation Council region and other water-stressed environments.

## 1. Introduction

*Phragmites australis*, commonly known as common reedgrass, is a typical wetland vegetation known for its aggressive invasiveness in regional ecosystems. This robust, herbaceous, perennial wetland plant plays a crucial role in wetland ecosystem services, such as carbon storage, nutrient cycling, and heavy-metal absorption [1]. This plant exhibits rapid growth potential, as it is capable of reaching heights of up to 5 m [1]. Once established, it rapidly forms dense monotypic stands through aerial seed dispersal and extensive rhizome networks [2]. *Phragmites australis* can quickly dominate invaded areas due to its fast growth rates [2], salinity tolerance [3], and exploitation of anthropogenic impacts on wetlands [4]. In particular, in North America, Phragmites is recognized as an aggressive invader in regional ecosystems [5]. Hence, to uphold the diversity and resilience of wetland ecosystems, it is crucial to regulate and manage the aboveground biomass (AGB) of common reed. An essential aspect of this management involves effectively estimating the AGB of the common reed. Despite extensive global research on wetland carbon dynamics, limited studies have quantified the biomass and carbon sequestration of *Phragmites australis* under hyper-arid conditions such as those in Oman. Furthermore, integration of destructive sampling with remote sensing for scalable carbon estimation remains underdeveloped in such environments.

On the other hand, *Phragmites australis* presents significant long-term carbon (C) reservoirs that accumulate atmospheric carbon at annual rates comparable to terrestrial forest types despite their limited global coverage [6,7]. These carbon stocks and exchanges in intertidal areas are collectively referred to as “coastal wetland blue carbon” [8]. Specifically, tidal marsh carbon stocks sequester carbon at average rates of 1–2 Mg C per hectare per year [9], although they are undergoing conversion to open water or other land cover types at global rates of 1–2% [10,11].

*Phragmites australis* plays a vital role in upholding the ecological balance of wetlands and offering various ecosystem services, including water purification, habitat provision, biodiversity conservation, and carbon sequestration [12,13]. Through photosynthesis, during its growth phase, *Phragmites australis* can assimilate significant amounts of carbon, thereby aiding in mitigating carbon emissions and promoting carbon neutrality [14]. Biomass, which refers to the dry weight of the living organic part of *Phragmites australis,* serves as a crucial indicator of its capacity for carbon sequestration [15]. In light of growing concerns about climate change due to excessive carbon emissions, there is a rising interest in estimating the biomass and carbon sequestration potential of wetland plants like *Phragmites australis*. This attention aims to support carbon management efforts and work towards achieving carbon neutrality [16,17,18].

The conventional method of measuring plant biomass involves on-site sampling at the plot level, a process that is known to be time-consuming, labor-intensive, and sometimes impractical due to challenges related to site accessibility [19]. In addition to field sampling, a growth dynamics model for vegetation has been devised to estimate biomass. These models are grounded in the eco-physiological processes of the plants they model, such as photosynthesis, respiration, and mortality. This approach enables a more frequent measurement of plant biomass, e.g., on a daily basis, providing a more detailed understanding of biomass variation and carbon sequestration capacity [20,21,22]. Asaeda and Karunaratne [21] were pioneers in establishing a growth dynamics model for *Phragmites australis*, focusing on key variables like shoot biomass, inflorescence, roots, old rhizomes, and new rhizomes. They used mathematical relationships to quantify plant growth by considering photosynthesis, respiration, mortality, and material translocation between organs, effectively reflecting the growth dynamics of *Phragmites australis* [14,21]. Through specific calibration and validation, this model has been employed in various case studies to explore *Phragmites australis* growth dynamics [14,23]. For example, ref. [14] studied the meteorological effects on *Phragmites australis* growth in the Scheldt estuary, while ref. [23] simulated biomass and carbon sequestration in a Mediterranean eutrophic lake. Environmental factors like latitude and nutrient availability have been parameterized in these models to simulate plant growth traits and their response to environmental changes, aiding in carbon management [20,24,25]. However, the development and application of these growth models depend on field biomass data for necessary calibration and validation [26].

Plants absorb and distribute both macro- and micro-nutrients within their tissues, facilitating the transfer of these nutrients among various plant organs. This internal cycling of nutrients is significant in wetland ecosystems. Notably, high-yield plants such as *Phragmites australis* can extract substantial quantities of nutrients from their surroundings on an annual basis. As a result, wetland plants are employed to mitigate nutrient levels in domestic, industrial, and agricultural wastewater streams [27,28].

A key challenge in applying standard biomass-estimation approaches to hyper-arid wetlands is phenotypic plasticity: plants growing under strong, spatially variable water and salinity stress can alter their height, tiller density, and stem architecture in response to micro-scale differences in resource availability, independent of their total biomass or carbon content. This plasticity means that simple morphological measurements, which are often reliable biomass predictors in temperate or well-watered systems, may not scale consistently with biomass under hyper-arid conditions, and that allometric equations calibrated on other species or environments may not transfer directly to a hyper-arid wetland grass such as *Phragmites australis*. This consideration motivates the approach taken in this study: rather than assuming a single field-measured predictor or an externally derived equation will hold, it is necessary to explicitly test multiple candidate predictors and approaches against destructively measured biomass and carbon content at this site.

Remote sensing is an advantageous method for monitoring vegetation. Various types of remote sensing data, such as optical images, light detection and ranging (LiDAR) point cloud data, and synthetic aperture radar (SAR) data, have been extensively utilized to quantify the aboveground biomass (AGB) of different plants [29]. Through establishing relationships between remote sensing data, like reflectance and vegetation indices, and actual biomass collected in the field, remote-sensing technology enables rapid and accurate estimation of AGB [30,31]. This technology proves particularly beneficial in wetland environments due to their remote and hard-to-access nature, as it covers vast areas [32]. Classifying wetland scenes from satellite imagery nonetheless remains methodologically challenging, since wetland vegetation typically presents complex, mixed spectral and spatial signatures that complicate accurate delineation from surrounding land cover [33]. For instance, ref. [34] demonstrated the capability of hyperspectral compact airborne spectrographic imager data to retrieve the AGB of *Phragmites australis* in the Zhangye National Wetland Park, China, while ref. [5] developed an empirical model for AGB retrieval of these vegetations in Dafeng, China. Therefore, biomass data acquired through remote sensing serves as a valuable resource for calibrating and validating plant growth dynamics models. There is a lack of an established framework to assess the significance of *Phragmites australis* (reedgrass) in carbon sequestration within the hyper-arid region including Oman. Hence, the main objective of this study was to evaluate the biomass and carbon dynamics of *Phragmites australis* in arid environments by addressing four key aspects: (i) quantifying aboveground biomass and carbon stock in destructively sampled plots, (ii) comparing aboveground biomass (AGB) estimates derived from dry biomass and total organic carbon (TOC) approaches, (iii) testing whether externally derived, height-based allometric equations transfer to arid wetland conditions such as Oman, and (iv) assessing the potential of remote-sensing techniques for scalable estimation of biomass and carbon sequestration.

## 2. Results

*Phragmites australis*, commonly known as the common reed, is a perennial grass species that is widely distributed in wetland ecosystems worldwide. It exhibits remarkable adaptability to various environmental conditions, including freshwater and brackish marshes, riverbanks, and disturbed habitats. *Phragmites australis* plays a significant ecological role in wetlands and offers various ecosystem services, including carbon sequestration and aboveground biomass production.

**Figure 1:**
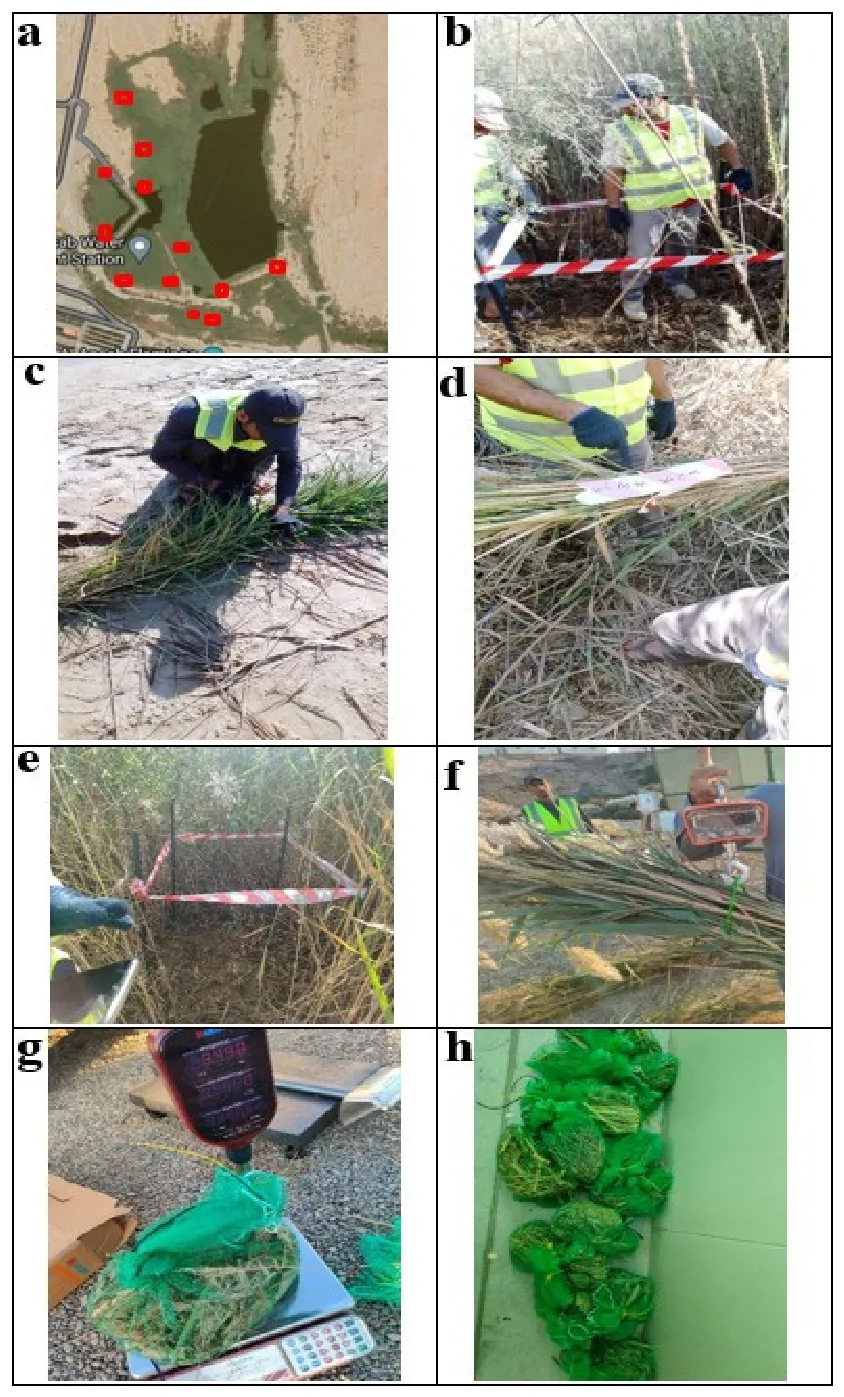
Destructive sampling of *Phragmites australis* (reed grass). (**a**) Site location of targeted plants; (**b**–**f**) cutting process of grass from targeted plots (~1 m × 1 m); (**g**) collection of fresh weight on site; (**h**) samples of 12 plots collected for lab-drying process and samples in drying room.

This study involved a meticulous process of collecting data on reed biomass using destructive sampling methods (Figure 1). The aboveground shoots of reedgrass were systematically collected from approximately 12 selected locations within the designated plant area to facilitate an in-depth analysis of carbon stock. The collection area for each sample encompassed a 1 × 1 square meter plot. To ensure accurate measurement, proper care was taken to obtain representative samples from various parts of the reedgrass population.

### 2.1. Carbon Stock Comparison

Upon collection, the reedgrass samples underwent a meticulous process of carbon stock analysis. The first step involved oven-drying the samples at 60–80 °C, following the methodology described by [35,36,37] and the IPCC, 2014 [9]; they were dried in an oven until a constant weight was achieved (dry weight). This process facilitated the removal of moisture and ensured the determination of the dry-weight biomass of the reedgrass shoots.

The concentrations of total organic carbon (TOC) in the reedgrass samples were quantified using a TOC analyzer, which is a widely recognized and reliable instrument for determining carbon content. This analysis allowed for precise measurement of the TOC levels, which is an important parameter for assessing the carbon stock.

The carbon stock of the reedgrass samples was determined by multiplying the TOC percentage by the corresponding dry sample weight. The calculated carbon content was then expressed in grams per square metre (g/m^2^) to estimate the carbon stock within the studied reedgrass ecosystem.

**Figure 2:**
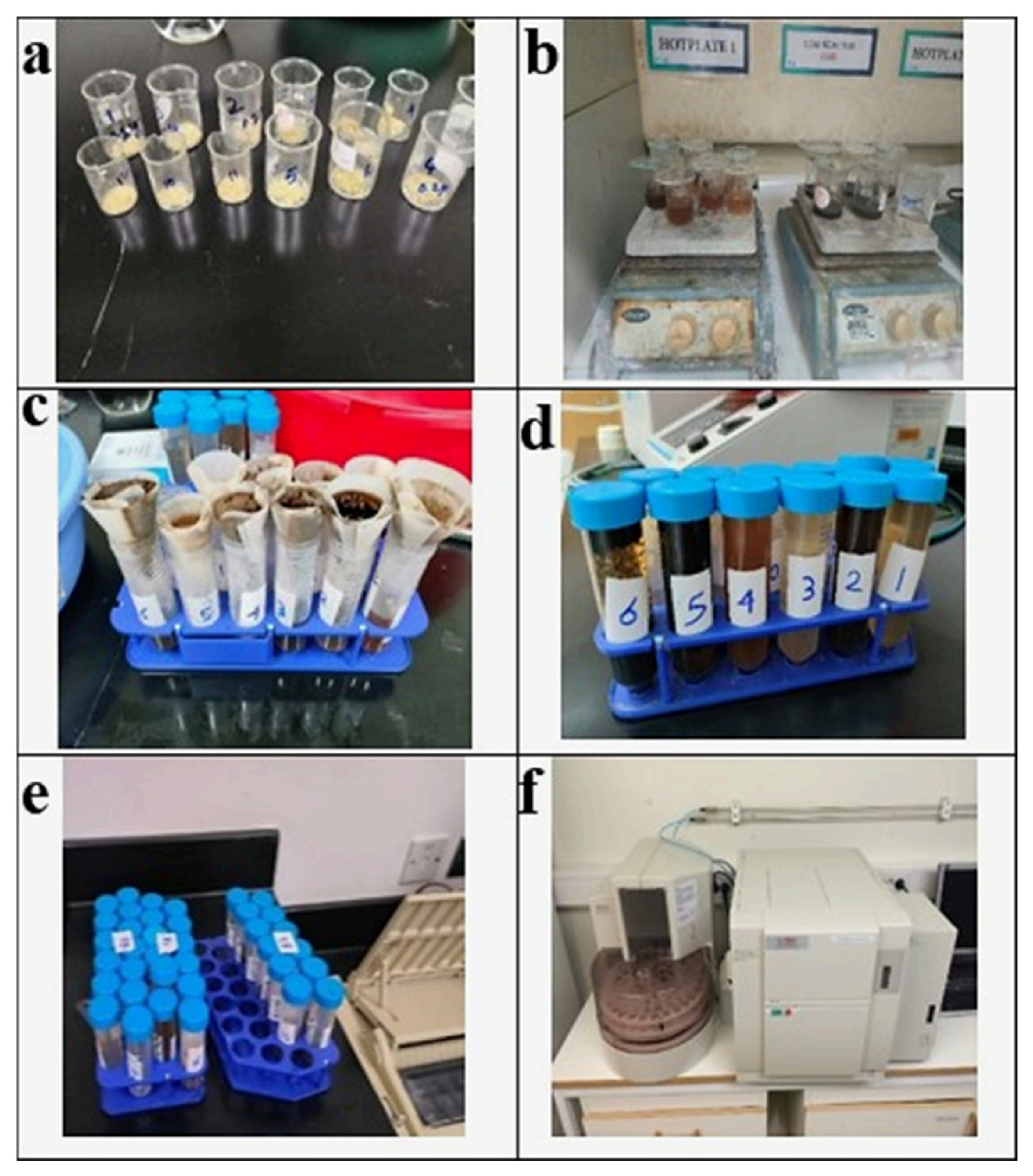
TOC of *Phragmites australis* (reedgrass) destructive sampling. (**a**–**f**): TOC calculation of 12 plots samples in 3 batches using a TOC-V analyzer.

The results are presented in a structured manner to highlight biomass distribution, carbon stock estimation approaches, model performance, and the integration of remote-sensing techniques for biomass assessment. See Figure 2 for a visual representation of the complete destructive site process employed in this study. The process encompasses several crucial steps, including pre-cutting measurements, comprehensive weight determination of all reedgrass plot components, systematic sample collection, precise acquisition of the fresh weight of the samples, and subsequent controlled drying of the samples within the controlled environment of the university drying laboratory. This visual depiction serves as a valuable guide, providing a comprehensive overview of the step-by-step procedures involved in the site process leading up to the drying phase. By incorporating this visual representation in our academic paper, readers can easily grasp the procedural flow and understand the meticulous approach taken to ensure accurate measurements and a consistent drying process.

The illustrated data pertain to various plot numbers within the designated site, detailing their dimensions, reed heights, reed count per plot, reed plot weight, as well as the fresh and dry weights of samples gathered from each plot. The plots are sequentially numbered from 1 to 12, each measuring 1 m by 1 m, and are systematically sampled surrounding the reed oasis area.

For clarity, four related but distinct variables are used throughout this section and are defined here: (1) fresh weight (kg/plot), which refers to the raw weight of harvested reed material recorded in the field before drying (Table 1); (2) dry weight (g), which is the weight of the oven-dried subsample used to derive the wet–dry ratio (Table 1); (3) aboveground biomass, AGB (kg/m^2^), which is the dry-mass-equivalent biomass per unit area, obtained either from the dry-weight ratio approach or converted from the TOC approach (Table 2); and (4) carbon stock (kg/m^2^), which is the carbon content per unit area, derived by applying the carbon conversion factor to AGB (dry-ratio approach) or measured directly via TOC analysis. The values quoted below are labeled explicitly with the corresponding variable and unit to avoid any ambiguity between these related quantities.

Aboveground biomass varied significantly across the 12 plots (Table 1), with reed height ranging from 2.2 to 4.3 m. Notably, tiller density did not correlate linearly with aboveground biomass (AGB), as Plot 1 exhibited the highest tiller density (214 tillers) but only a moderate raw fresh weight (3.7 kg/plot, equivalent to 1.81 kg/m^2^ AGB from the dry-ratio approach) compared to Plot 9 (73 tillers, 7.5 kg/plot fresh weight, equivalent to 3.91 kg/m^2^ AGB).

Furthermore, Table 2 presents data related to two different approaches for estimating carbon stocks in reed plots. The first approach is the dry weight ratio approach, which includes the reed wet–dry ratio. The reed aboveground biomass (AGB) is listed in kilograms per square meter (kg/m^2^) and is calculated based on the dry weight ratio; the reed carbon stock in kg/m^2^ is also calculated. The second approach is the TOC (total organic carbon) approach, which includes the TOC percentage in the collected sample plots.

For each plot, the values for the different parameters in both approaches were calculated. The reed wet–dry ratio ranges from 0.306 to 0.744, indicating the moisture content of the reed samples. The reed AGB from the dry weight ratio varies between 1.51 kg/m^2^ and 3.91 kg/m^2^, while the reed carbon stock from the dry weight ratio ranges from 0.75 kg/m^2^ to 1.95 kg/m^2^. In the TOC approach, the TOC percentage (expressed as a percentage of sample dry weight) ranges from 10.8% to 25.2%, representing the proportion of carbon content in the reed samples. The carbon stock from the TOC analyzer varies between 0.1933 kg/m^2^ and 0.6843 kg/m^2^.

This data allows for comparisons between the two approaches and the assessment of carbon stocks in the reed plots using different estimation methods. Further analysis could involve evaluating the consistency or discrepancies between the results obtained from the dry weight ratio approach and the TOC approach and discussing the implications for carbon stock assessments in reed ecosystems (Table 2).

Total organic carbon (TOC) method: The TOC method estimates biomass by determining the amount of organic carbon present in the plant material. This method involves collecting plant samples, drying them to remove moisture, and then analyzing the carbon content using laboratory techniques such as elemental analysis or combustion.

The TOC method provides an indirect estimation of biomass based on the assumption that a certain proportion of the plant’s organic carbon is derived from biomass. The actual proportion can vary depending on the plant species and environmental factors, so it is essential to calibrate the method for specific ecosystems.

Dry-to-wet ratio method: The dry-to-wet ratio method estimates biomass by determining the ratio of dry weight to wet weight of the plant material. It involves collecting fresh plant samples, weighing them immediately (wet weight), and then drying them in an oven until a constant weight is achieved (dry weight). The biomass is calculated by multiplying the wet weight by the dry-to-wet ratio.

The dry-to-wet ratio method assumes that the water content of the plant material is relatively constant, and the difference between wet weight and dry weight is mainly due to water loss during drying. This method can provide a quick estimation of biomass in the field, but it may introduce some errors if there are significant variations in water content among samples or if the water content changes during the drying process.

Both methods have their advantages and limitations, and the choice of method depends on the specific research objectives, available resources, and environmental conditions. It is always important to consider the specific characteristics of the plant species and ecosystem being studied and to validate the chosen method against ground truth measurements whenever possible.

**Figure 3:**
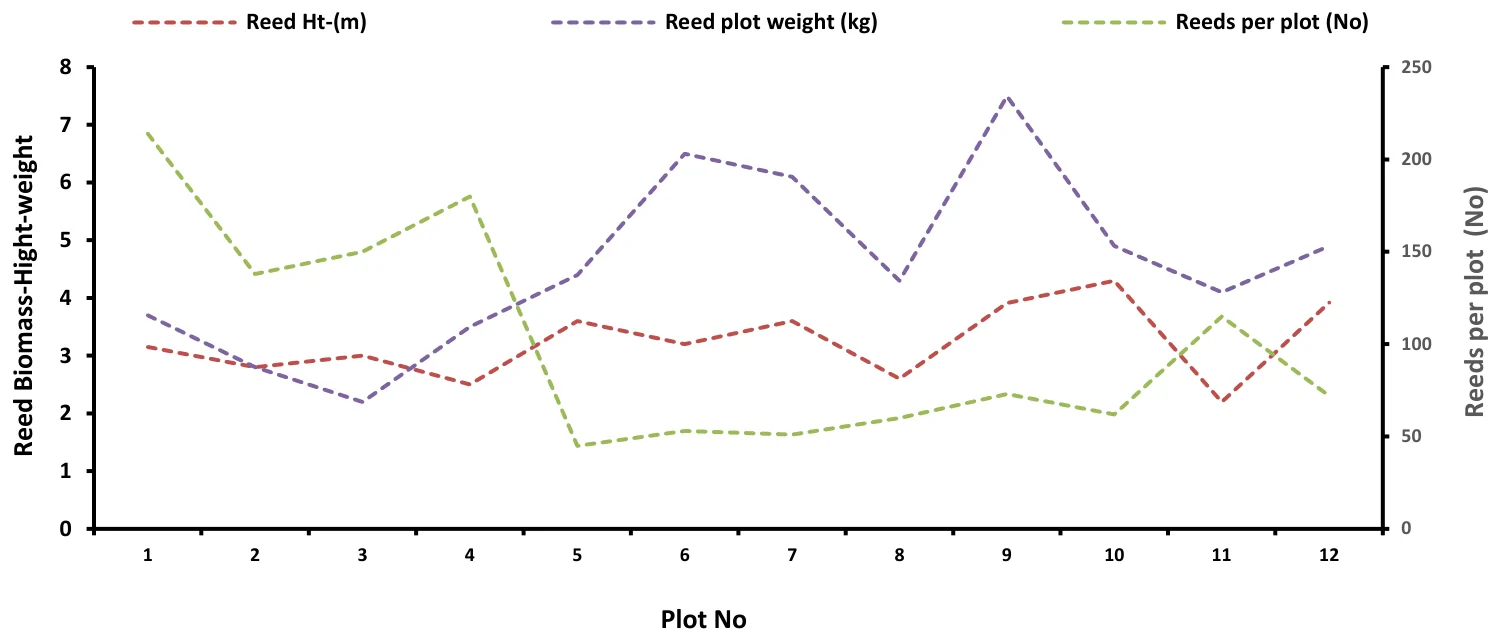
Destructive sampling of *Phragmites australis* (reedgrass): targeted plot number (position) versus fresh weight/plot (kg, left *y*-axis), reed height (m, right *y*-axis) and number of reeds per plot (No, secondary axis).

Figure 3 presents a visual snapshot of the destructive sampling process conducted within the reedgrass oasis, showcasing the 12 plots that were analyzed. The figure provides a visual representation of the in situ measurements taken at each plot, including the number of reeds present, the weight of the reed plot, and the height of the reeds. This visualization offers a concise overview of the collected data, enabling a comprehensive understanding of the distribution and characteristics of the reedgrass within the studied area. Figure 3 shows the spatial arrangement and quantitative measurements associated with the reedgrass plots, enhancing the clarity and comprehensiveness of our research findings.

**Figure 4:**
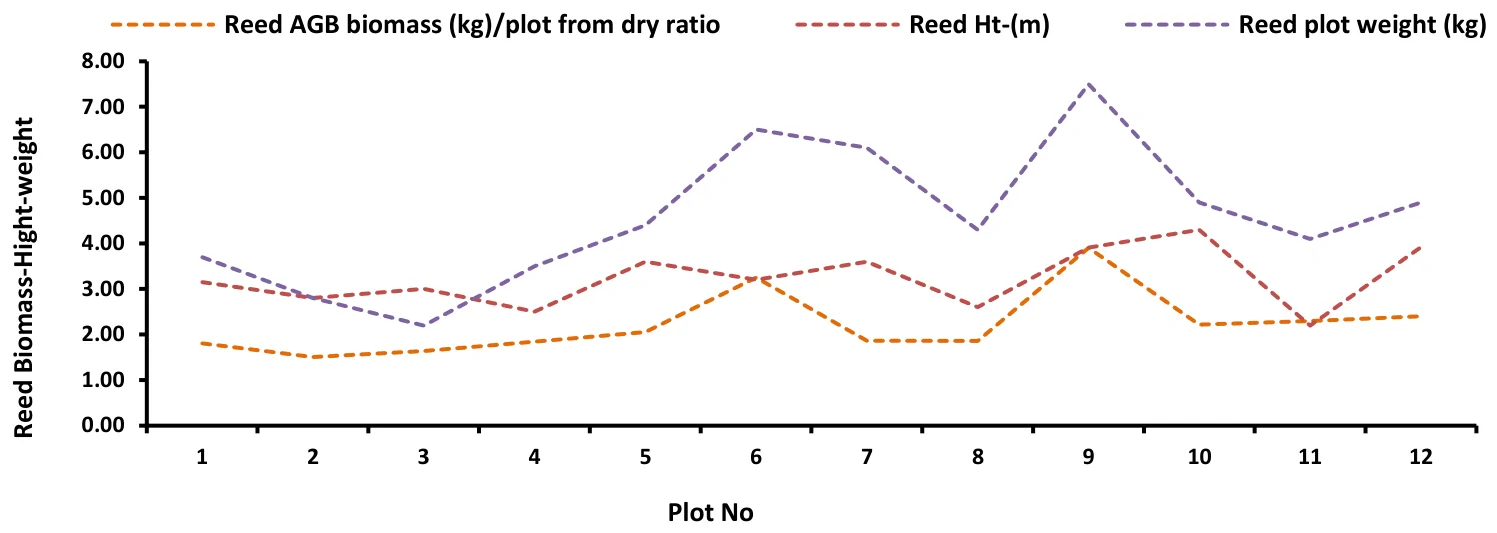
Destructive sampling of *Phragmites australis* (reedgrass): targeted plot number (position) versus reed AGB (kg/m^2^, left *y*-axis, dry-ratio approach), reed height (m) and reed plot fresh weight/plot (kg, secondary axis).

**Figure 5:**
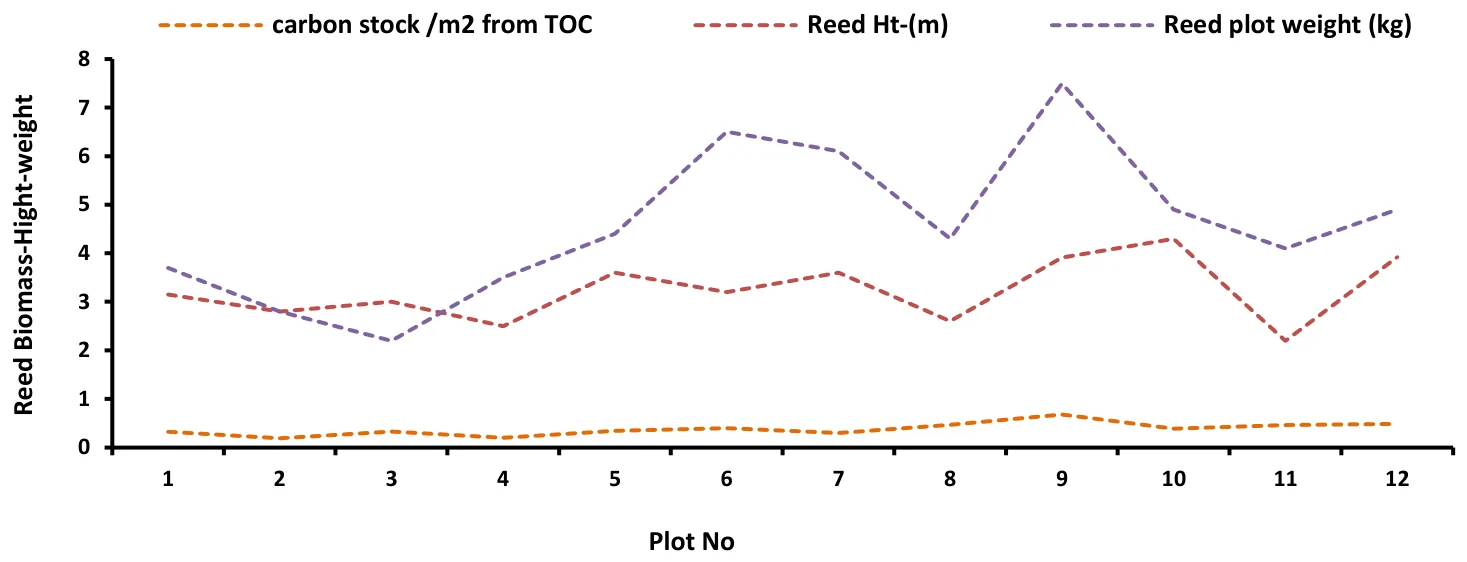
Destructive sampling of *Phragmites australis* (reedgrass): targeted plot number (position) versus reed AGB (kg/m^2^, left *y*-axis, TOC approach), reed height (m) and reed plot fresh weight/plot (kg, secondary axis).

Figure 4 and Figure 5 provide a comparative analysis of the aboveground biomass (AGB) of *Phragmites australis* across 12 plots within the oasis. These figures showcase the comparison between two distinct approaches used for calculating AGB, despite their differences in methodology. Interestingly, the results obtained from both approaches demonstrate a remarkable degree of similarity, indicating a high level of agreement. Furthermore, these findings align closely with previous studies conducted on *Phragmites australis*, as demonstrated in Table 3. The inclusion of Figure 4 and Figure 5 enables a visual representation of the AGB comparisons, reaffirming the consistency and reliability of our findings, while also highlighting the broader consistency with prior research on *Phragmites australis*.

**Figure 6:**
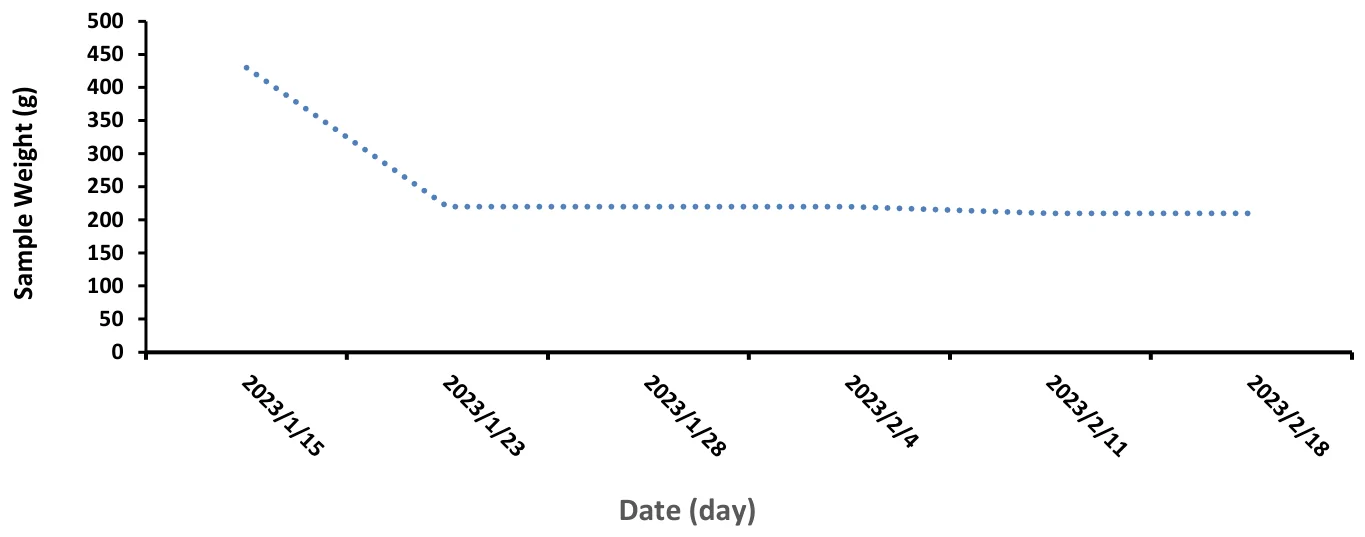
Destructive sampling of *Phragmites australis* (reedgrass): lab-calculated dry weight over days (out of 12 plots) versus samples’ fresh-dry (moisture loss) weight (g).

Figure 6 illustrates the relationship between the lab-calculated dry weight of the reedgrass samples and the corresponding fresh-dry weight (moisture loss) over a specific timeframe, measured in grams. The findings indicate a noticeable reduction in moisture content occurring over an approximate duration of 8 days, consistent with the high water content typical of non-woody, herbaceous tissue. This laboratory desiccation curve reflects the water content and drying behavior of harvested plant material and should not be interpreted as a measure of the ecosystem’s carbon sequestration capacity, which is assessed separately in this study via the dry-weight-ratio and TOC approaches (Section 2.1).

**Figure 7:**
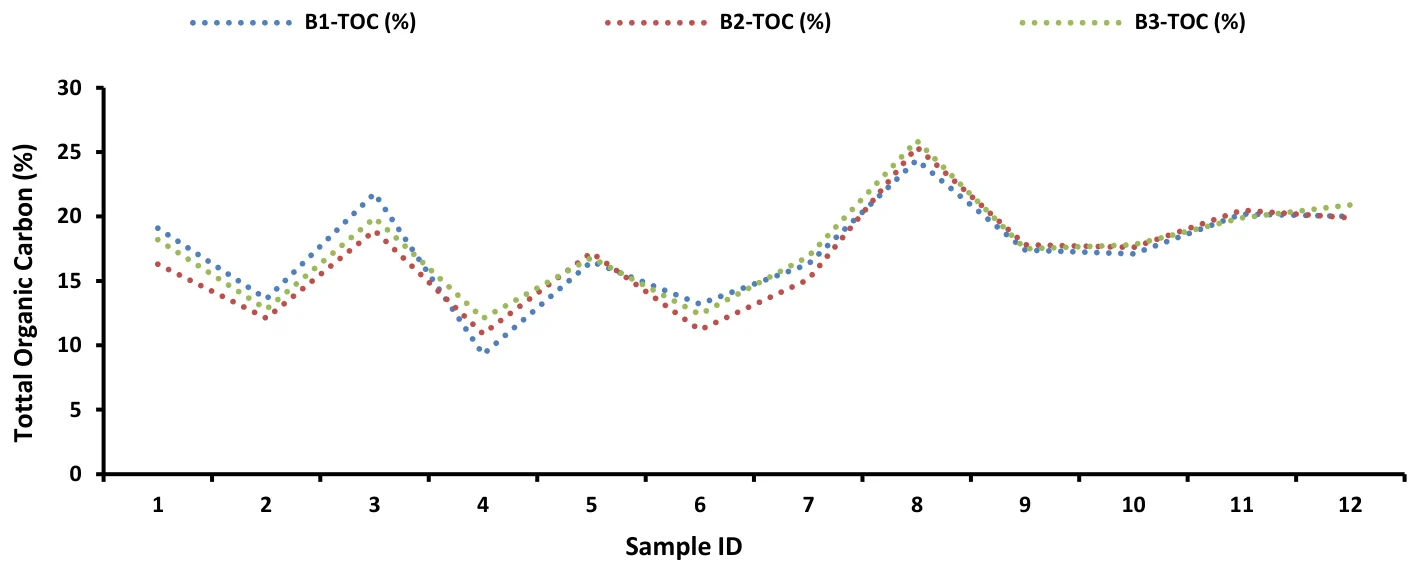
Destructive sampling of *Phragmites australis* (reedgrass): total organic carbon output of 12 plots using TOC-V analyzer (No) versus three batches’ TOC (% of sample dry weight).

Figure 7 presents the values of three sample batches comprising 12 plots each, in relation to the output of total organic carbon expressed as a percentage (%). The figure provides an overview of the correlation between the sample batches and their corresponding plots. Notably, the results reveal a consistent correlation value across all batches and similar plots.

**Figure 8:**
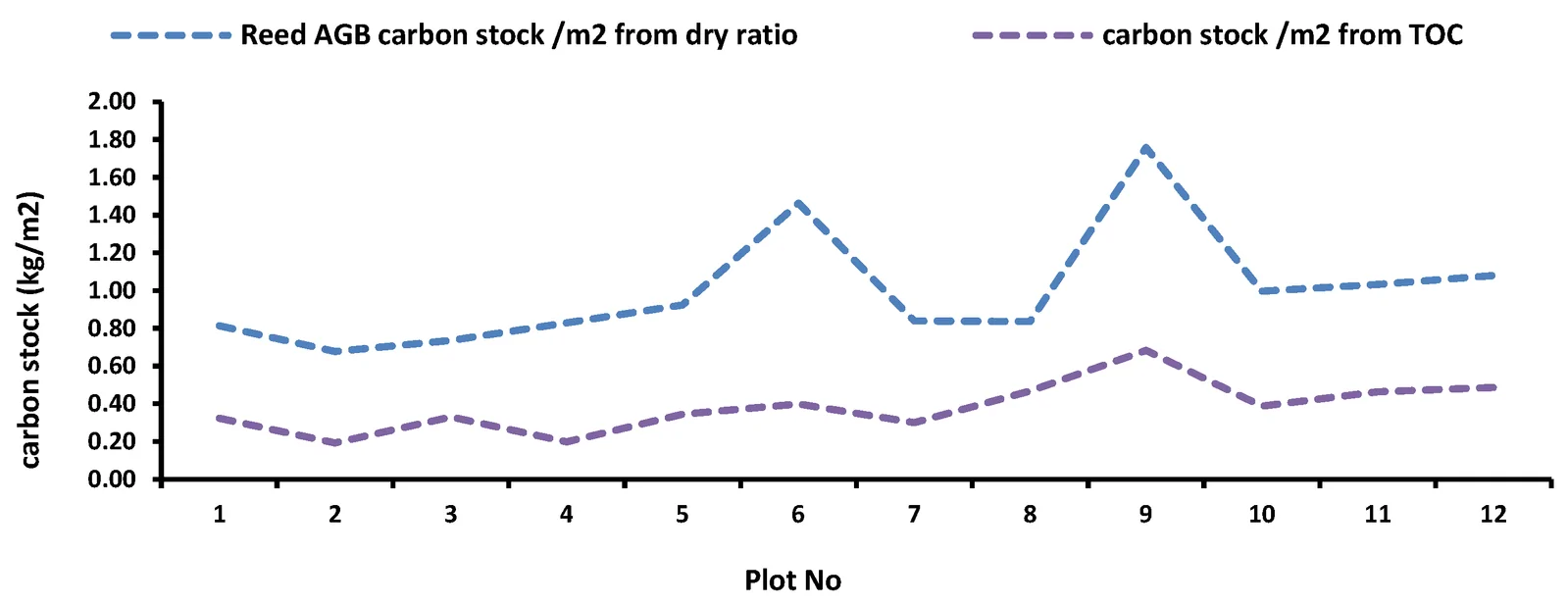
Destructive sampling of *Phragmites australis* (reedgrass): comparison of targeted plots (No) versus carbon stock (kg/m^2^) from the two-way approach.

Figure 8 illustrates the carbon stock estimates across the 12 plots using two different approaches: the dry/fresh ratio method and the total organic carbon (TOC) approach. The two approaches do not agree closely: the TOC-based carbon stock is consistently and substantially lower than the dry-weight-ratio-based estimate, by a factor of roughly three on average across the 12 plots (0.38 kg/m^2^ versus 1.11 kg/m^2^; Table 2). This is an expected consequence of how each approach is calculated—the TOC approach measures organic carbon content directly, whereas the dry-weight-ratio approach applies a fixed conversion factor (Section 4.3) that was developed for woody biomass and is not calibrated for herbaceous wetland species—rather than evidence against either measurement. The size of this gap, the species-specific literature values against which it can be judged, and its likely sources are examined in the Discussion.

### 2.2. Model Performance

The central aim of the reed model’s evaluation fitting was to thoroughly assess the predictive precision of the ultimate selected models when estimating individual aboveground biomass (AGB) for reeds. To achieve this objective, the fitting dataset was employed, which contains relevant measurements and data pertaining to the target reed population. The use of this dataset allowed for a meticulous evaluation of the models’ performance and their capacity to accurately estimate the AGB of the reed oasis.

**Figure 9:**
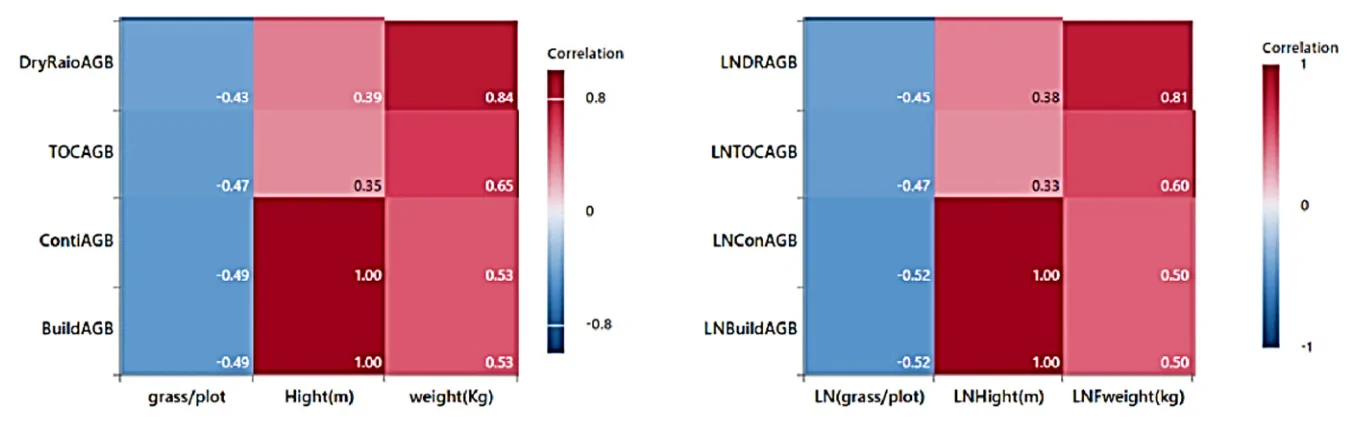
Matrix plot correlation of *Phragmites australis* (reedgrass) novel models of AGB versus plant measured variables.

**Figure 10:**
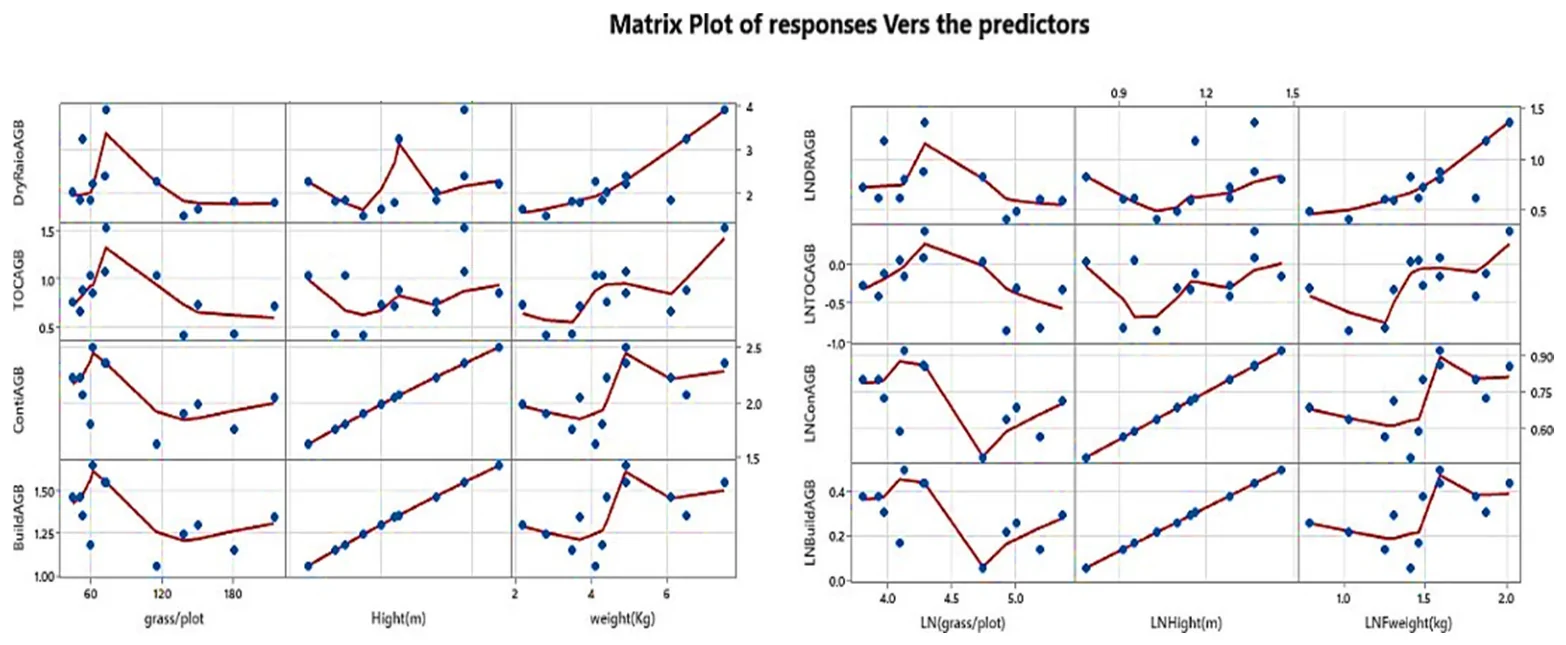
Matrix plot correlation of *Phragmites australis* (reedgrass) novel models of AGB versus plant measured variables in 2D.

**Figure 11:**
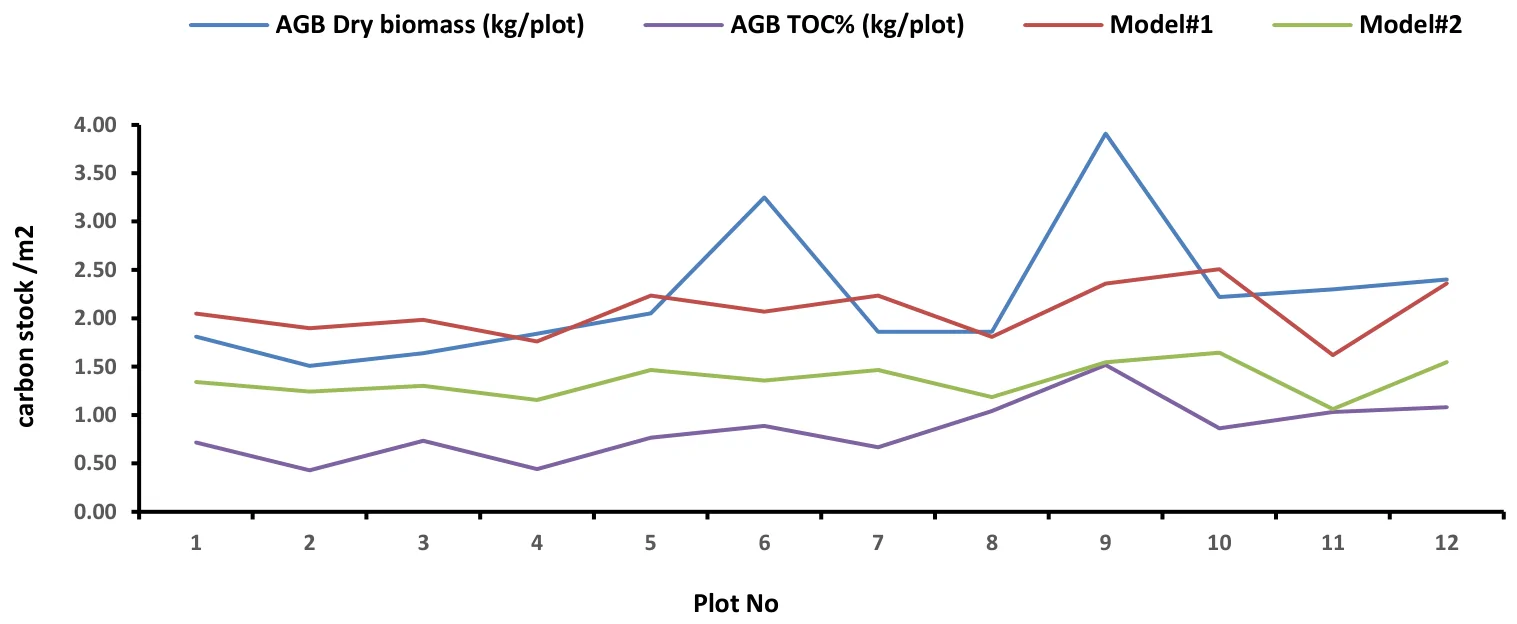
Correlation of *Phragmites australis* (reedgrass) AGB versus plant measured plots of destructive sampling along fitted study equations and calculated biomass approach.

The first approach involved utilizing the dry-to-fresh ratio method, which entails quantifying biomass based on the weight ratio of dried reed samples to their corresponding fresh weight. The second approach was the total organic carbon (TOC) method, which involves estimating AGB by measuring the total organic carbon content of reed samples (Figure 9, Figure 10 and Figure 11). In addition to these two approaches, the study also applied a height-based allometric equation from [48], noting an important caveat: that equation was originally developed for shrub species, not wetland grasses, and its transferability to *Phragmites australis* has not been independently validated; it is used here as an external benchmark for comparison against the dry-to-fresh ratio and TOC methods, not as a validated predictive model for this species.

Comparing the dry-to-fresh ratio and TOC methods against the Conti et al. benchmark allowed the relative strengths and limitations of each approach to be assessed directly using the same dataset, providing a basis for recommending the most defensible method for estimating aboveground biomass in this system.

**Figure 12:**
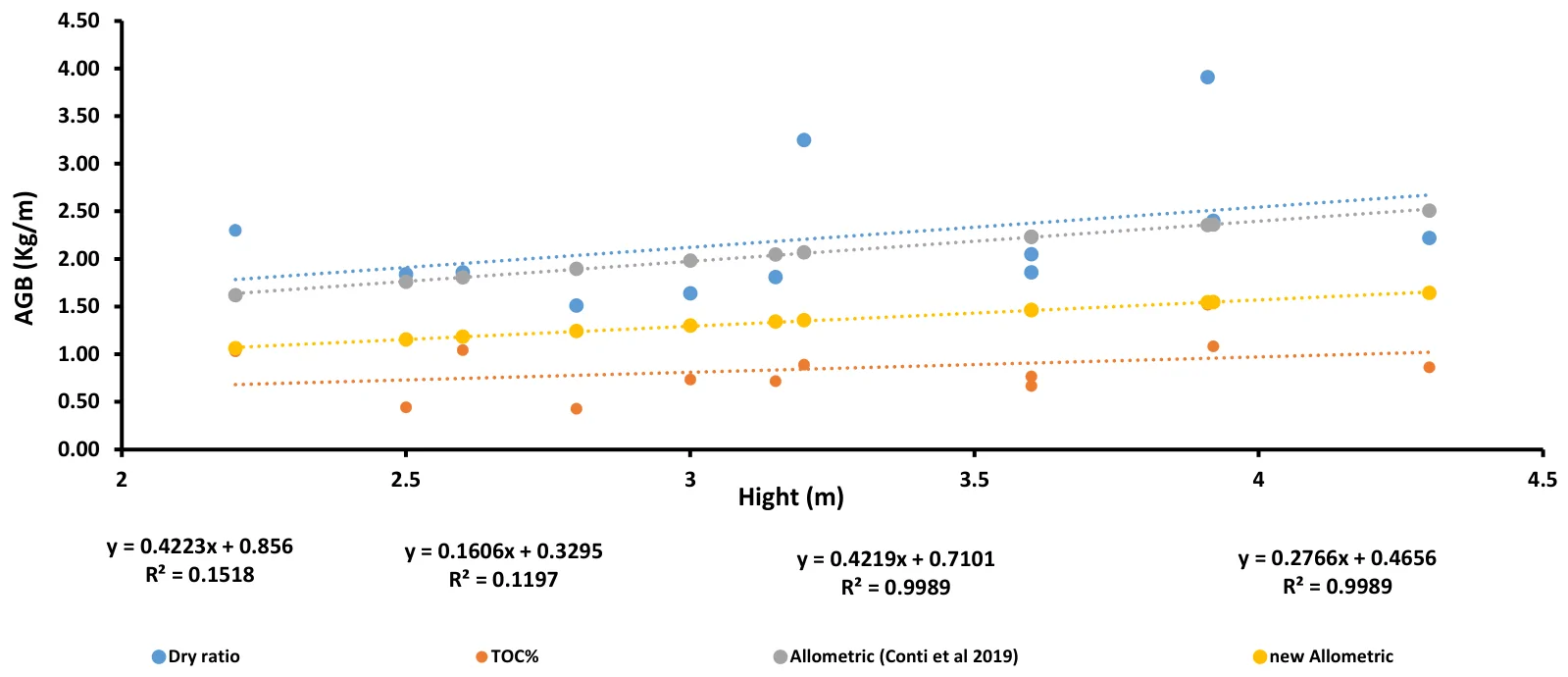
Reed height plotted against destructively measured AGB (Table 2) and against the AGB values predicted by the two height-based allometric equations (Model#1, from [48]; Model#2, this study’s recalibration of that equation). The near-perfect fit of both model-predicted series reflects their mathematical dependence on height rather than independent validation; see the Discussion for the correlation between height and the destructively measured AGB values.

**Figure 13:**
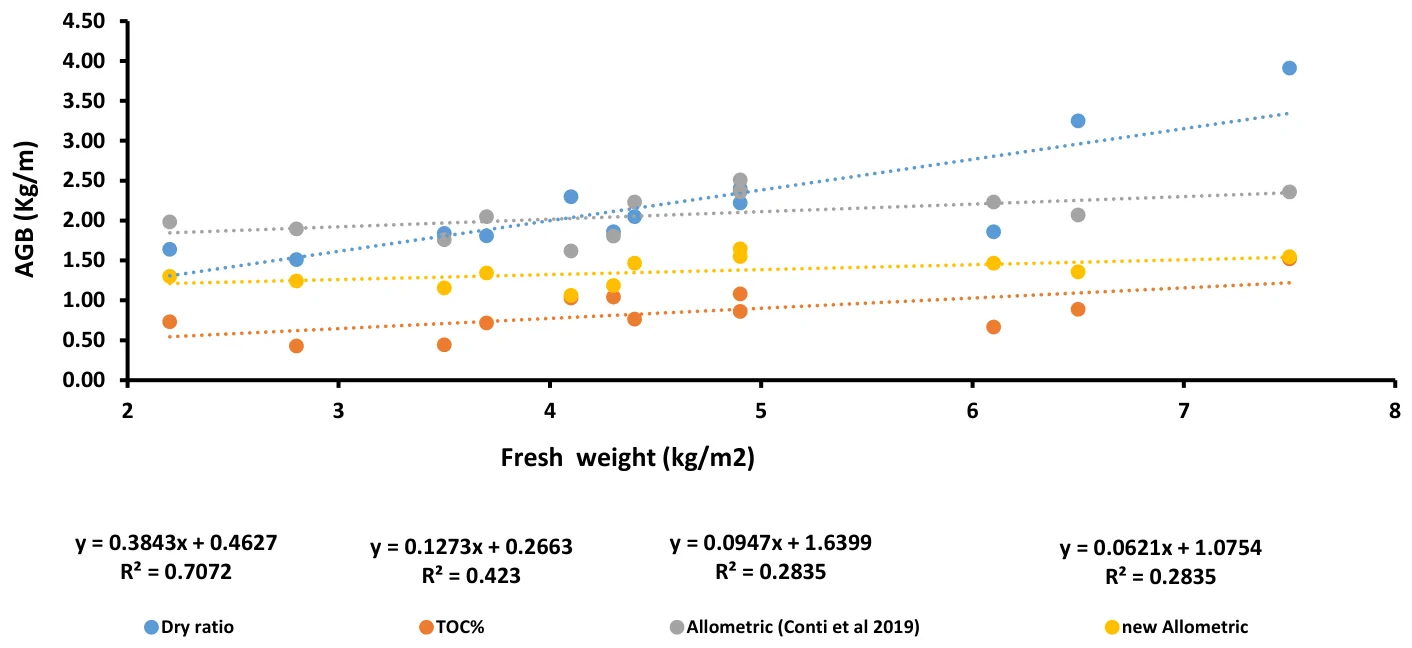
The evaluation of correlated studies [48] and measured AGB along destructive reeds versus the weight predictor.

**Figure 14:**
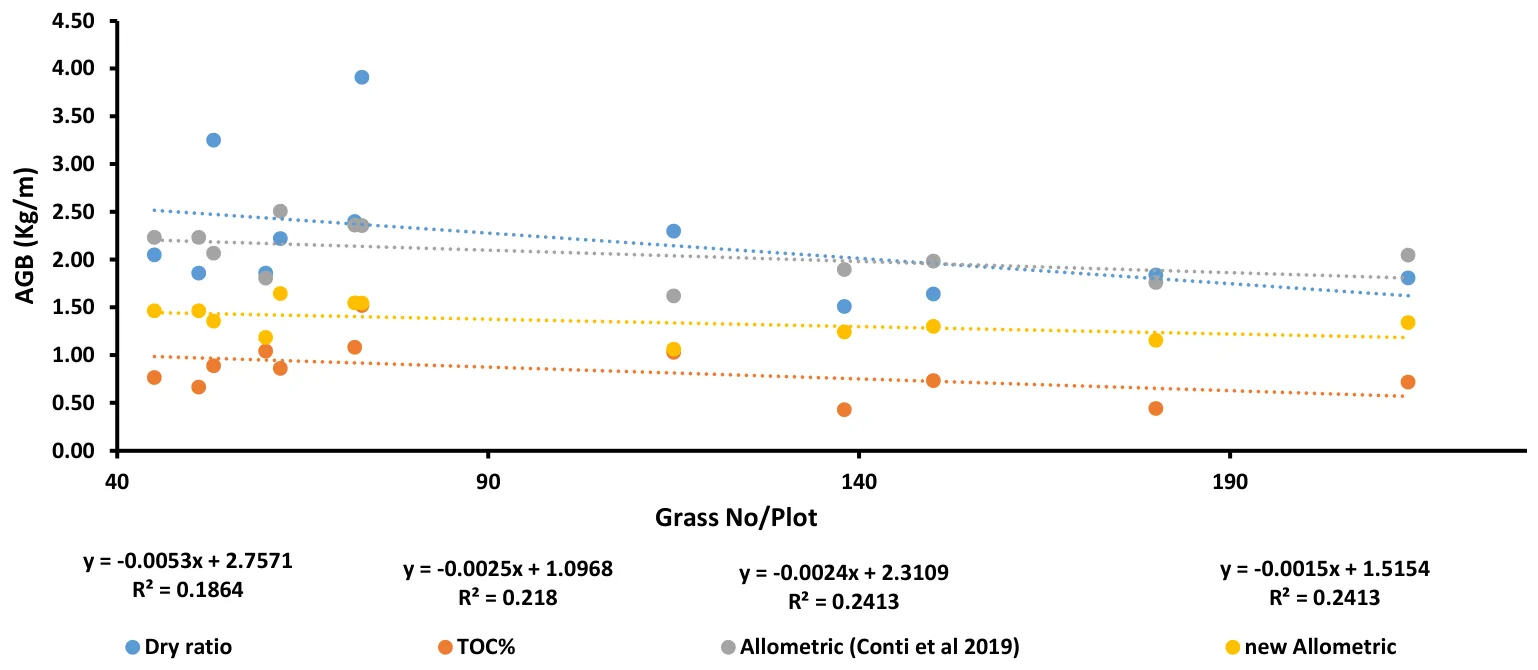
The evaluation of correlated studies [48] and measured AGB along destructive reeds versus the number of reeds-per-plot predictor.

The subsequent matrix presents a comprehensive analysis of the correlation between the responses, focusing on aboveground biomass (AGB) or its natural logarithm (LN(AGB)), and the calculated predictors for reeds. The predictors examined include reed height (measured in meters), fresh weight (measured in kilograms per square meter), and the number of reeds stands per unit area of one square meter (Figure 12, Figure 13 and Figure 14). The matrix results unveil a significant correlation between the [48] study and the correlated regression equation, particularly concerning the reed height predictor. This finding indicates a strong alignment between the outcomes of the [48] study and the regression equation, specifically when estimating reed height.

However, the calculated aboveground biomass (AGB) obtained from both approaches showed notably weak correlations with the predictors of reed parameters (height, weight, and reed number). The exceptions to this were the calculated dry AGB, which demonstrated a commendable correlation with the reed weight predictor only. These correlation findings provide valuable insights into the efficacy of the applied methodologies for estimating aboveground biomass in reeds. The notable correlation with reed weight in the context of calculating dry AGB suggests a potential avenue for enhancing biomass estimation accuracy. Nonetheless, the observed weak correlations between the predictors and the calculated AGB highlight the complexity of the biomass estimation process for reeds. This detailed analysis aims to contribute significant knowledge to the field of vegetation biomass estimation and improve ecological modeling practices.

In the pursuit of novel models for estimating the aboveground biomass (AGB) of *Phragmites australis* (reedgrass), equations were integrated from a prior study conducted by [48]. These regression model equations focused on destructive correlation of *Phragmites australis* (reedgrass) data, as documented in Table 4. Subsequently, stepwise regression was applied to a dataset encompassing measurements obtained from 12 distinct plots of *Phragmites australis* (reedgrass). A comprehensive evaluation of the newly devised models was undertaken, utilizing prominent indicators such as R2 and *p*-value to assess their efficacy.

Integral to the model construction, the test included essential reed variables, namely height, the weight of fresh reeds, and the number of reeds per plot. This strategic incorporation of these variables served to enrich the analytical framework and enhance the model’s predictive capacity. Among the various models explored for AGB estimation in *Phragmites australis* (reedgrass), two height-based logarithmic models stood out as candidates for further evaluation: Model#1, applied directly from the published allometric equation of [48], which was developed for shrub species rather than wetland grasses, and Model#2, a recalibration of that equation’s intercept to align with this study’s TOC-based carbon estimates, while retaining the same height-scaling slope. Because both equations compute AGB as a direct mathematical function of height, each shows a strong internal correlation with reed height by construction; this reflects consistency with their own input variable rather than independent validation against the destructively measured AGB values collected in this study.

**Figure 15:**
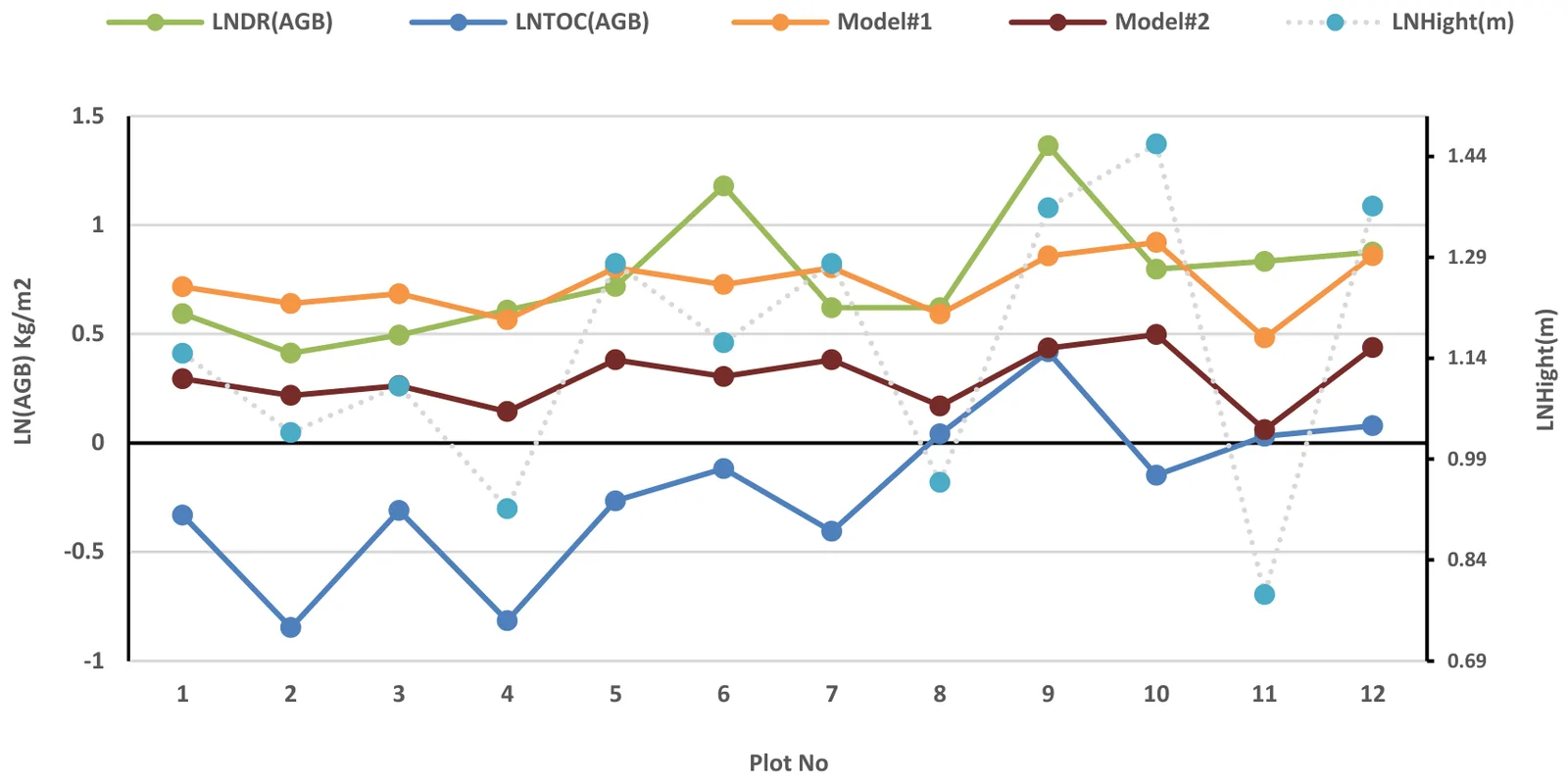
Correlation of *Phragmites australis* (reedgrass) novel models of AGB Logs versus the Log reeds’ measured height variable.

Within the fitting dataset, the AGB values produced by Model#1 were in closer numerical agreement with the calculated values from the harvested samples of the fresh-to-dry approach than Model#2, as depicted in Figure 15 (Table 4). As with all comparisons in this section, this reflects relative within-sample fit, not an independently validated predictive advantage; the cross-validated assessment of both models is reported separately in Table 5 and Section 2.3.

**Figure 16:**
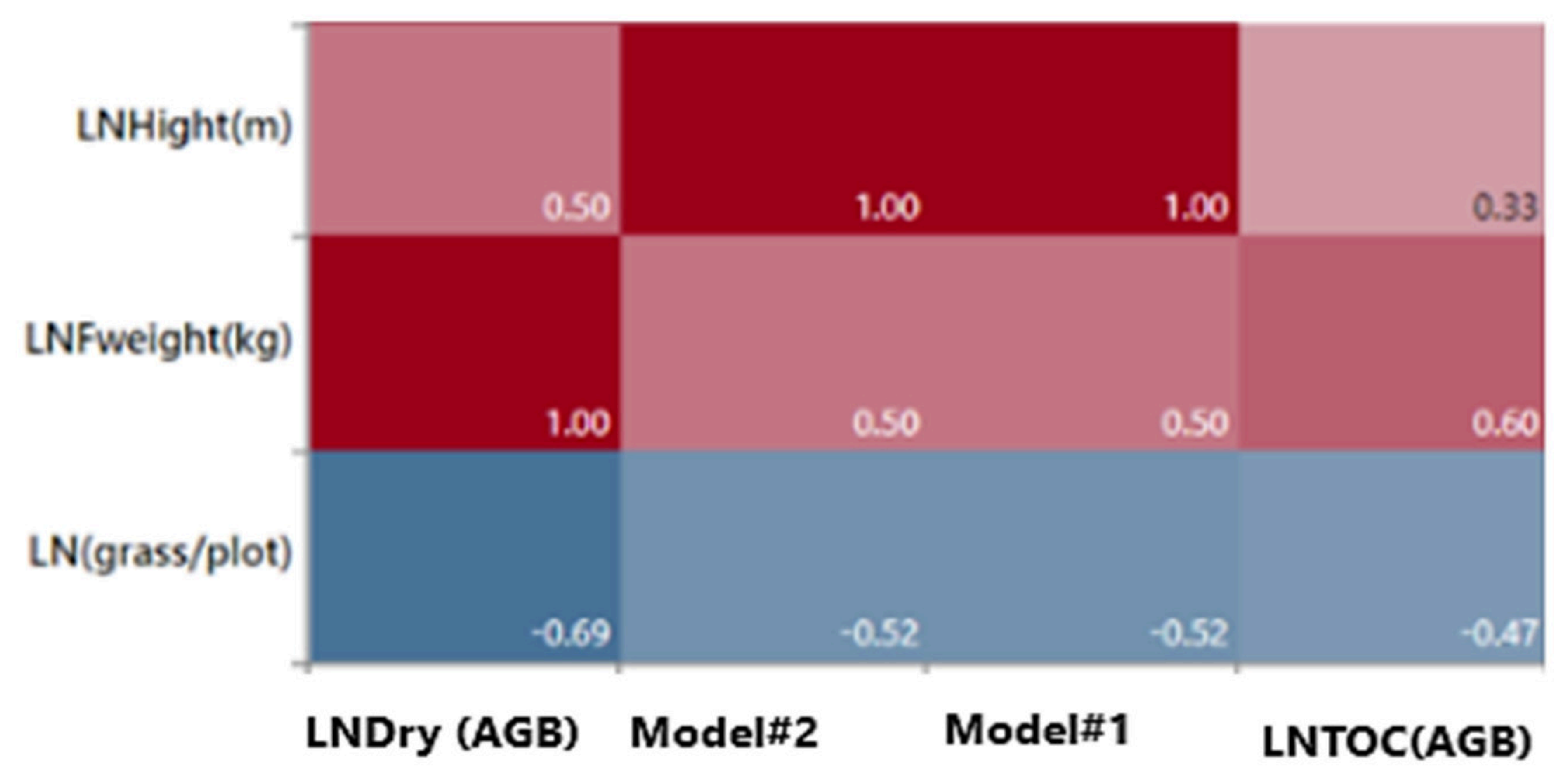
Matrix plot correlation of *Phragmites australis* (reedgrass) novel models of AGB Logs versus Log plant measured height variable.

Because Model#1 and Model#2 are both fitted to values falling between the fresh-to-dry and total organic carbon (TOC) calculation approaches, their AGB estimates for all plots fall between these two reference approaches by construction, as shown in Figure 15 and Figure 16. This positioning is a property of how the models were fitted, not evidence of improved estimation accuracy for *Phragmites australis* (reedgrass); on standard within-sample fit indicators, Model-2 shows less favorable MAE, MAPE, RE, and RMSE values than Model-1.

Table 5 reports the final regression equations fitted for estimating aboveground biomass (AGB) in *Phragmites australis* oasis environments. By evaluating correlation indicators such as MAE, MAPE, R2 and RMSE, Model#1 shows a smaller within-sample fitting error than Model#2; this comparison describes how closely each equation reproduces the dataset used to fit it, not a validated predictive advantage of either equation for *Phragmites australis* (reedgrass) AGB, given the cross-validated performance reported in Table 6 and Section 2.3.

Table 5 displays a comprehensive overview of our novel regression model devised for the estimation of aboveground biomass (AGB) within *Phragmites australis* (commonly known as reedgrass) populations. This model is the result of a meticulous statistical correlation regression analysis conducted across specific targeted locations. Drawing insights from prior research endeavors and on-the-ground measurements, these models incorporate a spectrum of variables and anticipated outcomes. They thus serve as invaluable tools for both practitioners and researchers seeking precise quantification of aboveground biomass within these plant communities. Furthermore, these models adopt a holistic approach by encompassing both total organic carbon and dry biomass methodologies.

Through this study, this model contributes to the evolving comprehension of carbon sequestration dynamics and biomass assessment methodologies across diverse ecological contexts. Such insights are pivotal for advancing ongoing initiatives in climate change research and ecological modeling endeavors.

The performance evaluation of the two height-based models (Model-1, Model-2; Section 2.2) against the destructively measured dry-biomass and TOC-based carbon-stock values is shown in Table 6. As with Table 7, the MAE, RMSE, and MAPE are reported on the back-transformed (real AGB or carbon-stock, kg/m^2^) scale rather than the log-transformed regression scale, so that error magnitudes are directly interpretable. For dry biomass, both models show weak explanatory power (Model-1: R^2^ = 0.10; Model-2: R^2^ = −1.52, indicating a fit worse than simply using the sample mean), with Model-1 showing smaller absolute and percentage error (MAE = 0.450 kg/m^2^, RMSE = 0.636 kg/m^2^, MAPE = 18.0%) than Model-2 (MAE = 0.861 kg/m^2^, RMSE = 1.064 kg/m^2^, MAPE = 35.1%).

For TOC-based carbon stock, both models were recalculated against the corrected TOC values (Section 2.1) using the same real-space method, with the height-derived AGB prediction converted to carbon using the same 0.50 factor applied elsewhere in this study. Both models perform substantially worse than the sample mean (Model-1: R^2^ = −26.0; Model-2: R^2^ = −5.3), with correspondingly large percentage errors (Model-1: MAE = 0.655 kg/m^2^, RMSE = 0.672 kg/m^2^, MAPE = 200.8%; Model-2: MAE = 0.298 kg/m^2^, RMSE = 0.324 kg/m^2^, MAPE = 97.3%). A strongly negative R^2^ is a stronger and more interpretable statement of model failure than a near-zero one: it indicates that the height-based prediction is actively worse than simply reporting the average measured value for every plot. This is consistent with our broader finding that TOC content is a compositional property of plant tissue with no expected mechanistic dependence on height, and we do not recommend either model for predicting TOC-based carbon stock from height in this system; Model-1 shows comparatively lower error for dry-biomass prediction, while neither model is suitable for TOC-based prediction.

The poor fit of the TOC-based models (Table 6) is not, on its own, a defect in the study: TOC% is a compositional laboratory measurement of organic carbon content and is not mechanistically linked to the morphological predictors (height, tiller density, and fresh weight) used in these regressions, so a near-zero or negative R^2^ is the expected outcome of testing this relationship rather than an anomaly. Therefore, we do not recommend the TOC-based allometric models as predictive tools for this dataset. Their value lies instead in independently verifying the carbon-fraction estimate (Table 2) and in flagging cases where the dry-weight-ratio approach may be biased by inorganic carbonate content in these alkaline soils.

### 2.3. Remote Sensing Validation

**Figure 17:**
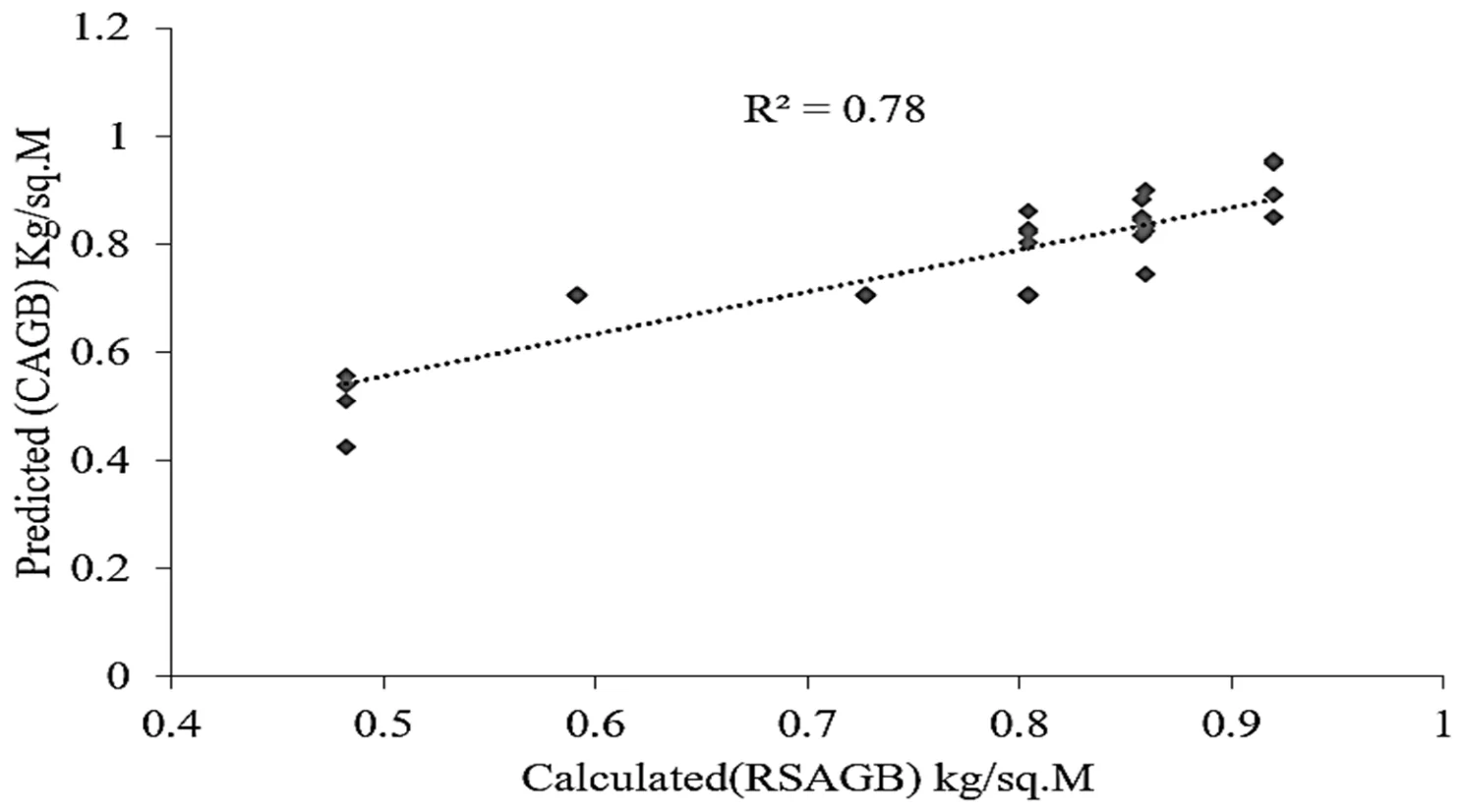
Destructive (Obs) to Model-1 performance fitness chart.

This investigation involved a comprehensive evaluation of spectral reflectance and vegetation indices (VIs) for reed plants across multiple model combinations. As summarized in Table 7 and visually shown in Figure 17, both Model 1 and Model 2, which incorporate satellite imagery, achieved an in-sample R^2^ of 0.78 for remote sensing aboveground biomass (RSAGB) when compared against a height-derived AGB proxy, referred to here as calculated aboveground biomass (CAGB). However, as detailed below, this figure does not reflect a validated relationship with destructively measured biomass: it does not survive cross-validation, and its target variable is itself derived from height rather than measured directly. The scope of what is validated below is also limited to the biomass-prediction step itself: the upstream Support Vector Machine (SVM) classification used to delineate reed cover from the satellite imagery (Section 4.6) was not independently accuracy-assessed, and its potential contribution to error is not captured by the cross-validation reported here. The following paragraphs present this analysis in full.

Model-1 and Model-2 were both derived using the same vegetation index; the two models differ in the allometric equation applied to convert the index value to AGB, which is why they achieve a similar R^2^ while differing in absolute prediction error (see below). In addition to the individual spectral bands, including red (R), green (G), blue (B), near-infrared (NIR), red edge (RE), and deep blue (DB), a set of vegetation spectral indices was used to characterize vegetation properties and assess their relationships with aboveground biomass. The vegetation indices included chlorophyll red edge (ChL_RE), green–red vegetation index (GRVI), normalized difference red edge index (NDRE), normalized difference vegetation index (NDVI), red–blue ratio index (RBRI), and red edge simple ratio/red edge spectral index (RE_SER). The Pléiades Neo satellite image was acquired on 28 August 2023, while field destructive sampling commenced on 11 September 2023, corresponding to a 14-day interval between image acquisition and field sampling.

This 14-day gap warrants a sensitivity discussion, since *Phragmites australis* can add substantial height and biomass within two weeks under favorable conditions. However, three factors limit the likely impact of this interval on the present results. First, both the image acquisition and field sampling occurred at the end of the summer growing season (late August to mid-September), when *P. australis* growth rates in this hyper-arid system are expected to be near their seasonal minimum due to sustained high temperatures and evapotranspirative water stress, rather than during the rapid spring growth flush. Second, the interval is short relative to the full growing season, and any systematic growth occurring uniformly across all plots would primarily shift the absolute regression intercept rather than the relative ranking of AGB among plots, which is what the vegetation-index correlations in this study depend on. Third, and most importantly, the cross-validation analysis presented below shows that the satellite predictors do not achieve a validated relationship with measured AGB regardless of this temporal offset; the 14-day gap is therefore unlikely to be the limiting factor behind that result, though we cannot rule out that it contributes some additional, unquantified noise. Eliminating this gap entirely in future work, by scheduling image acquisition and destructive sampling on the same day, remains good practice and is recommended for any follow-up study at this site.

The Model-1 and Model-2 regressions reported in Table 7 use all six vegetation indices together with the six individual spectral bands (12 predictors) fitted to n = 32 sampled points (eight independent plots, each sampled at four locations), a ratio of fewer than three observations per predictor and, at the plot level, fewer than one observation per predictor. A further methodological issue affects the interpretation of Table 7: its target variable is the aboveground biomass value predicted by the height-based equations (Model#1/Model#2, Section 2.2) rather than the destructively measured AGB itself. Because these equations compute AGB directly from height, and height alone was already shown (Figure 12; Section 2.2) to correlate only weakly with true destructively measured biomass, Table 7’s R^2^ = 0.78 measures agreement with a height-derived proxy, not with ground-truth biomass.

To assess the satellite predictors’ relationship with actual biomass, we refitted the regression directly against the destructively measured AGB values for the eight plots with satellite coverage and evaluated it using plot-level leave-one-out cross-validation (holding out all four sampled locations from a given plot together, so that no information from a held-out plot would leak into model fitting). Using the full 12-predictor set, the in-sample R^2^ was 0.59, but the plot-level leave-one-out Q^2^ was −2.42—far worse than the sample mean. We then exhaustively tested every one-, two-, and three-predictor combination from the same 12 candidate variables (286 combinations) against the destructively measured AGB values, selecting for the best possible leave-one-out performance. No combination achieved a positive Q^2^; the best-performing combination reached only Q^2^ = −0.48. The conclusion is that, with only eight independent plots carrying satellite coverage, this dataset cannot support a validated predictive relationship between the tested vegetation indices or spectral bands and destructively measured AGB, regardless of which or how many predictors are used. Table 7 is retained to document the original analysis and its R^2^ = 0.78 in-sample fit to the height-derived proxy, but this figure should not be read as evidence that the satellite predictors reliably estimate biomass at this site; a larger, independently sampled set of plots with satellite coverage would be required to properly evaluate this approach.

For completeness, Table 7 reports MAE, RMSE, and MAPE for the original height-derived-proxy analysis on the back-transformed AGB scale (kg/m^2^) rather than the log-transformed regression scale used in the original calculation, so that error magnitudes are at least internally interpretable; this correction does not address the target-variable issue described above. On this basis, Model-1 and Model-2 perform similarly to one another (R^2^ ≈ 0.78, MAPE ≈ 5.4% for both), which is expected since both are built from the same vegetation index and differ only in the allometric equation used to convert it to AGB—this indicates that the two equations behave similarly, not that either reliably predicts real biomass, which the plot-level cross-validation above shows they do not.

**Figure 18:**
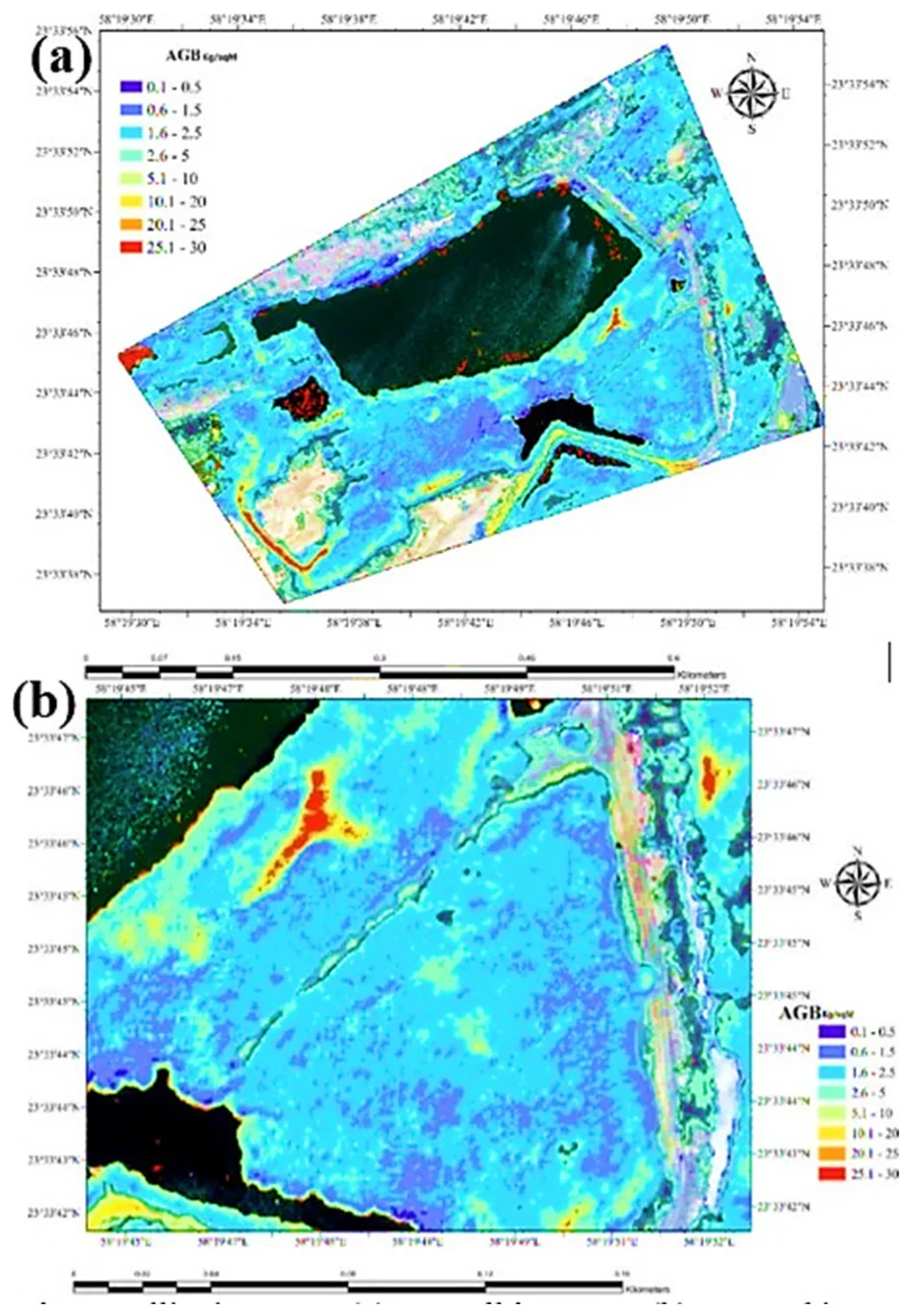
RSAGB estimated for reed plants using satellite imagery. (**a**) Overall imagery; (**b**) zoomed-in imagery.

Detailed maps illustrating RSAGB for reedgrass vegetation were constructed utilizing satellite imagery. These maps were instrumental in visualizing and depicting the distribution patterns of RSAGB across the reedgrass plants. As depicted in Figure 18, the overlay of RSAGB information was superimposed onto the identified locations of the detected reed plants at a pixel-based resolution.

This overlaying technique facilitated a visual representation highlighting the relationship between RSAGB distribution and the specific spatial arrangement of detected reed plants within the satellite imagery. The resulting map provided a comprehensive insight into the correlation between RSAGB patterns and the presence and distribution of reed plants, offering a detailed understanding of the biomass distribution dynamics across the reedgrass site.

**Figure 19:**
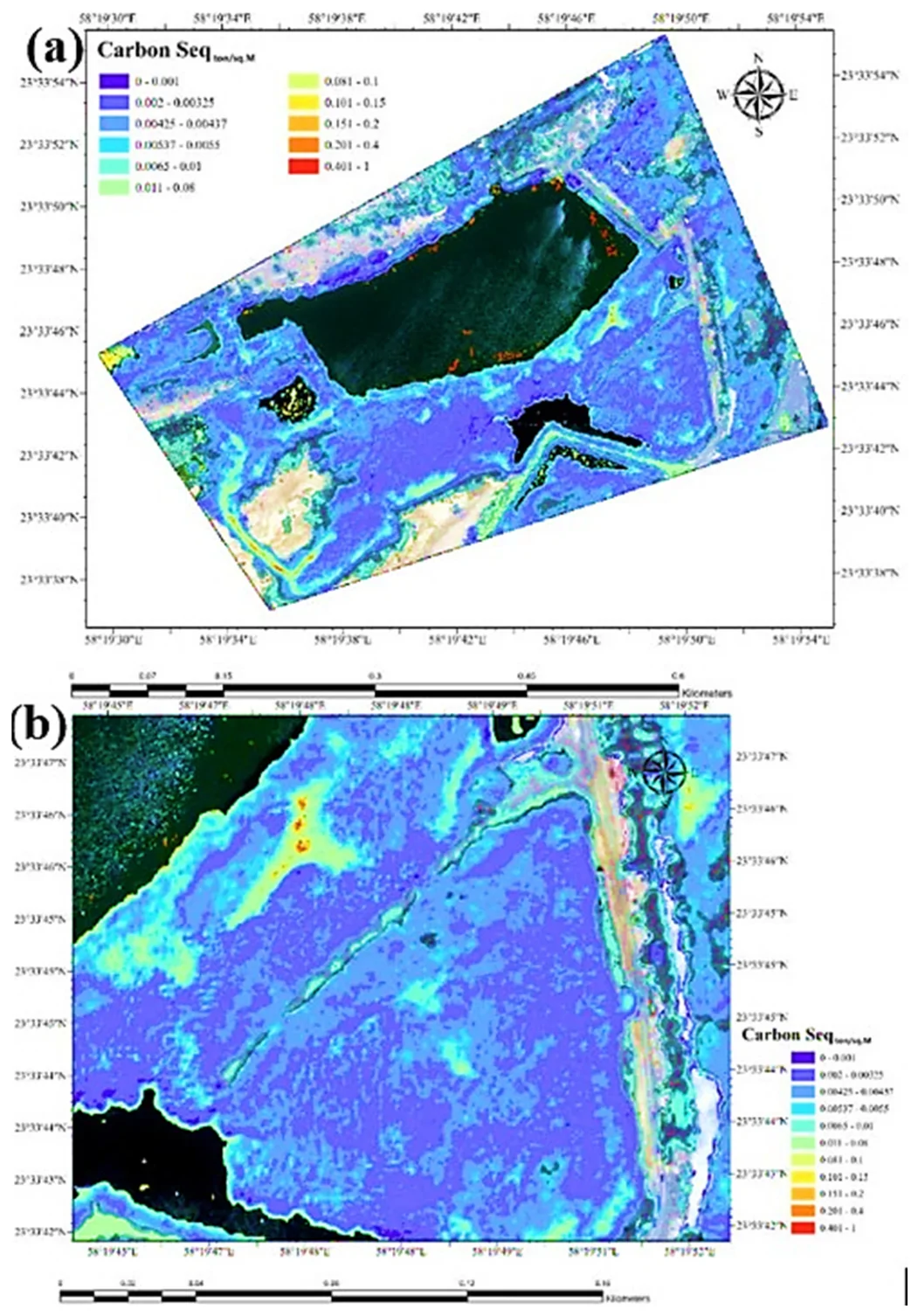
RS-Carbon-Seq. estimated using satellite imagery for the reed plants. (**a**) Overall imagery; (**b**) zoomed-in imagery.

In addition, intricate maps delineating carbon sequestration areas were meticulously crafted using satellite data in conjunction with machine learning algorithms (RS-Carbon-Seq.). These maps were specifically designed to visualize and analyze the distribution patterns of carbon sequestration areas derived from the RS-CarbonSeq methodology. As illustrated in Figure 19, an overlay technique was employed, showcasing the RS-Carbon-Seq. information superimposed onto the identified locations of detected reed plants at a pixel-by-pixel resolution. This visual representation provided a comprehensive depiction of the relationship between the delineated areas of carbon sequestration as per RS-CarbonSeq and the specific spatial arrangement of identified reed plants within the satellite imagery. This mapping approach facilitated a detailed exploration of the correlation between carbon sequestration zones and the distribution patterns of reed plants, thereby contributing to an enhanced understanding of the interplay between carbon sequestration and the presence of reed plants within the studied area.

## 3. Discussion

This study provides a rigorously validated, integrated assessment of biomass and carbon sequestration dynamics for *Phragmites australis* in a hyper-arid artificial wetland in Oman. Combining destructive sampling with remote sensing exposed the specific methodological challenges that complicate carbon accounting in highly heterogeneous arid ecosystems, and demonstrated concretely why they must be addressed before predictive models from either approach can be trusted here.

The carbon stocks observed in this study, ranging from 0.19 to 1.95 kg/m^2^ (equivalent to 1.9 to 19.5 Mg C/ha), demonstrate that *P. australis* maintains a measurable aboveground carbon stock even under extreme water stress; it should be stressed from the outset that this figure reflects aboveground biomass only, and excludes belowground rhizome and root carbon, which in Phragmites-dominated systems can substantially exceed the aboveground pool and is addressed further as a limitation in the Conclusions. This standing stock is not directly comparable to reported annual sequestration or productivity rates (e.g., 1–2 Mg C per hectare per year for intertidal blue carbon marshes [8], or the Mg ha^−1^ yr^−1^ productivity values listed in Table 3), since a stock (a quantity present at one point in time) and a flux (an annual rate of accumulation) answer different questions and require multi-season data to relate to one another; Table 3 therefore reports standing-stock comparisons separately from productivity-rate comparisons. Within the standing-stock comparisons, the Al Ansab values fall within the range reported for other Phragmites-dominated and salt-marsh systems (Table 3), suggesting that artificial wetlands constructed for wastewater treatment in arid zones concurrently function as significant carbon sinks, adding a critical climate-mitigation dimension to their well-documented water purification services [12,13].

A pivotal finding of this research is that none of the simple field-measured biometric parameters tested—plant height, tiller density, and fresh weight—showed a strong standalone correlation with destructively measured aboveground biomass (AGB) at this sample size. Height correlated only weakly with measured AGB (R^2^ = 0.15), a magnitude comparable to the localized allometric models incorporating tiller density, fresh weight, and dry biomass (R^2^ < 0.16); see Figure 12 and its caption for the distinction between this measured correlation and the near-perfect fit of the height-based allometric equations to their own input variable. This outcome is nonetheless informative for arid ecology. In temperate regions, tiller density is often a reliable predictor of biomass [15]; however, in hyper-arid environments, plants exhibit high phenotypic plasticity in response to micro-variations in water and salinity availability, and no single field-measured biometric variable captured this variability well enough to serve as a reliable standalone predictor. As evidenced by Plot 1 (214 thin tillers; moderate biomass) versus Plot 9 (73 robust tillers; high biomass), high tiller density does not equate to high carbon storage under arid stress, and height alone does not resolve this either. The experiment also tested whether remote sensing, which integrates canopy-level spectral information rather than a single morphological measurement, could overcome this limitation; applying the same plot-level cross-validation standard against destructively measured AGB (Section 2.3) produced a consistent result across both approaches, defining a clear, quantitative sampling-scale threshold that this system requires for either type of predictor to be trusted operationally. Standard ground-based allometric equations developed in temperate or mesic environments [5] are likely inadequate for arid wetlands; the larger, multi-plot dataset with satellite coverage identified by this benchmark is the direct next step toward establishing whether remote sensing can meet it.

This correlation was re-examined in light of the small sample size (n = 12). Figure 12 plots, against reed height, both the destructively measured AGB values (Table 2) and the AGB values predicted by the two height-based allometric equations described in Section 2.2 (Model#1, from Conti et al. [48]; Model#2, this study’s recalibration of that equation). These two series are not equally informative: because Model#1 and Model#2 compute AGB directly as a function of height, their predicted values are mathematically constrained to track height almost perfectly (R^2^ = 0.999), independent of how well the underlying equation predicts real biomass—this reflects the equations’ internal consistency with their own input variable, not an independent validation. The scientifically meaningful test is the correlation between height and the destructively measured AGB values themselves, which is markedly weaker (R^2^ = 0.15). Height alone is therefore not a strong standalone predictor of measured aboveground biomass in this dataset, and the near-perfect R^2^ obtained from the height-based allometric equations should not be read as evidence that height predicts real biomass well.

The sample size of n = 12 destructive plots reflects the physical and ethical constraints of this study design: destructive sampling was restricted to preserve the integrity of the constructed wetland (an active wastewater-treatment facility), each plot required both oven-drying and TOC analysis in triplicate batches, and accessibility within the reed oasis limited the number of locations that could be safely and non-disruptively sampled. This sample size limits the statistical power of the plot-level allometric models; it therefore presents these models as a pilot-scale, hypothesis-generating result that should be validated with a larger and/or multi-season dataset before being applied predictively elsewhere.

The failure of traditional plot-level allometry in this study underscores the need for alternative approaches to carbon accounting in arid wetlands, and remote sensing remains a reasonable candidate to explore in future work. In this study, however, vegetation indices and spectral bands derived from satellite imagery did not provide a validated predictor of destructively measured AGB: an initial in-sample fit (R^2^ = 0.78) reflected agreement with a height-derived proxy rather than measured biomass, and when tested directly against measured AGB using plot-level leave-one-out cross-validation, no combination of the twelve available predictors—including the best of 286 combinations tested—achieved a positive, generalizable fit (Section 2.3). This is consistent with the well-documented risk of overfitting regression models with many candidate predictors on small field-sampled datasets, compounded here by the limited number of independent plots (n = 8) carrying satellite coverage. Remote sensing in principle captures the spectral reflectance of the canopy, which is inherently linked to chlorophyll content, leaf area index, and vegetation structure—factors that collectively represent “plant vigor” better than simple physical counts, and satellite-based approaches have shown efficacy in other wetland environments [29,34]. Confirming whether this holds at the Al Ansab site will require a substantially larger and independently validated dataset than the present study provides [30,31].

The comparison between the dry-weight-ratio and total organic carbon (TOC) approaches reveals a substantial and mechanistically explicable discrepancy: the TOC approach yields an average carbon stock of 0.38 kg/m^2^, roughly one-third of the 1.11 kg/m^2^ obtained from the dry-weight-ratio approach using the generic IPCC 0.50 carbon factor. Part, but not all, of this gap narrows once a species-specific factor is used in place of the generic IPCC default: Silan et al. [38] report a directly measured carbon fraction of 0.43–0.45 (mean ≈ 0.44) for *Phragmites australis* specifically, consistent with the 0.40–0.45 range Verma et al. [39] report across 117 herbaceous species; applying this 0.44 factor to this study’s measured AGB gives an intermediate carbon-stock estimate of 0.98 kg/m^2^ (Table 2). This value is a modest, defensible refinement of the 1.11 kg/m^2^ IPCC-default figure, but it remains far closer to 1.11 kg/m^2^ than to the 0.38 kg/m^2^ obtained directly from TOC analysis—so switching to a better literature-derived carbon fraction resolves only a small part of the discrepancy, and something else must account for the remainder. We considered two further explanations. The first was a units or basis error: the 17.3% mean TOC figure is calculated, as described in Section 2.1, as a percentage of oven-dry sample mass rather than fresh mass; we re-checked this calculation against the underlying laboratory records and confirmed that it is not a fresh-weight artefact. The second was a mineral (inorganic) carbon contribution: the TOC analyzer used here quantifies total carbon released on combustion, and in the absence of an acid-pretreatment step to remove inorganic carbonates, any calcium or magnesium carbonate adhering to or incorporated within the harvested reed tissue—plausible given the alkaline, carbonate-rich soils and irrigation water typical of this constructed wetland—would be counted as part of the measured TOC signal, which, if anything, would inflate rather than deflate the TOC-based figure relative to true organic carbon; it therefore cannot by itself explain why the TOC estimate is lower than the dry-weight-ratio estimate, but it does mean the 17.3% figure should be treated as an upper bound on organic carbon content rather than a precise value, and we flag it as a priority for confirmation by acid pretreatment (e.g., HCl fumigation) prior to TOC analysis in any follow-up sampling at this site. We therefore read the remaining gap primarily as reflecting genuine physiological differences between *Phragmites australis* tissue at this hyper-arid, saline site and the generic literature values it is being compared against, rather than as a methodological artefact, while acknowledging that this cannot be fully disentangled with the present dataset. To resolve this explicitly rather than leave three figures in parallel, where a single carbon-stock value for this site is required, we recommend the TOC-based estimate of 0.38 kg/m^2^ as the primary, directly measured figure. The dry-weight-ratio values of 1.11 kg/m^2^ (0.50 IPCC default) and 0.98 kg/m^2^ (0.44 species-specific factor [38,39]) are retained throughout this manuscript only as secondary, upper-bound estimates, reported for comparability with the substantial body of prior literature (Table 3) that applies carbon factors in this range; neither should be read as an independent confirmation of the TOC result, since all three draw on the same underlying dry-biomass measurements and differ only in the conversion factor applied. We recommend that future carbon-stock estimates for this and similar herbaceous wetland species be based on directly measured, acid-pretreated organic carbon content rather than generic woody-biomass conversion factors.

From a management perspective, these findings have practical implications for arid regions like Oman. Artificial wetlands, such as the Al Ansab site, are often established for waste management and biodiversity; this study suggests that, with continued monitoring, such sites may also be quantifiably valued for climate mitigation. The timing and intensity of vegetation management (e.g., mowing or harvesting) is itself a lever on wetland carbon outcomes: in other wetland systems, submerged-plant mowing timing has been shown to substantially alter both greenhouse-gas emissions and carbon sequestration, with mowing in mid-growing-season reducing CO2, CH4, and N2O fluxes while maintaining biomass accumulation [49]. Management-oriented remote sensing monitoring, of the kind demonstrated here, could similarly be used to schedule harvesting to maximize carbon retention in *P. australis* stands, though this specific mechanism was not tested in the present study and would require dedicated experimental work. Decision-makers should recognize that the ecosystem services provided by *P. australis* extend beyond local habitat provision to active atmospheric carbon removal. Moving forward, integrating field-inventory data with satellite-derived vegetation indices—rather than field-measured height alone—offers a promising basis for carbon monitoring at this site; extending this framework across the Gulf Cooperation Council (GCC) region’s other constructed wetlands would require validation at additional sites and across multiple seasons, which this single-site, single-season study does not provide.

## 4. Materials and Methods

**Figure 20:**
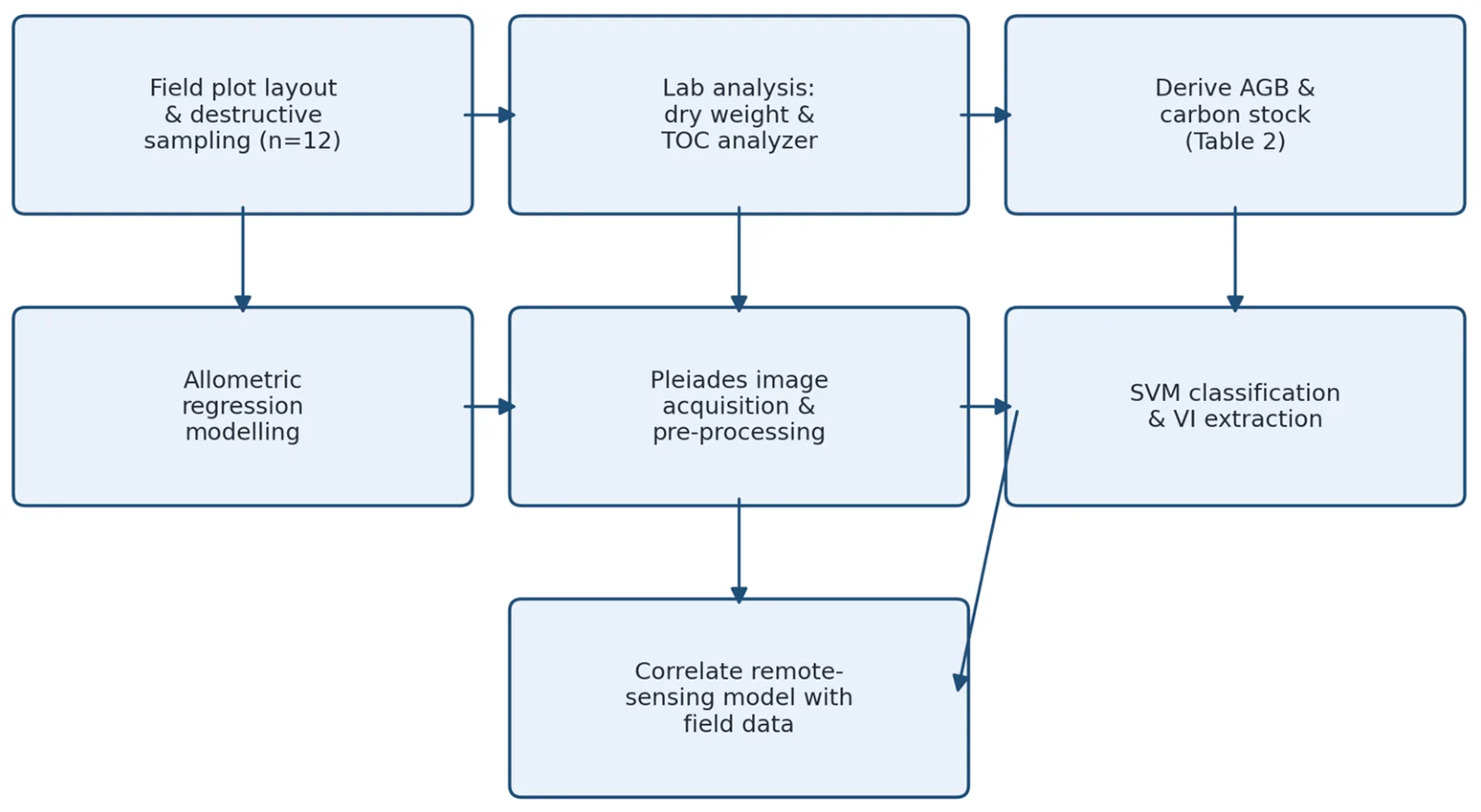
The overall workflow of the study methodology, from field destructive sampling and laboratory analysis through allometric regression modeling and satellite-based remote sensing to the correlation of the two approaches.

Figure 20 summarizes the overall workflow followed in this study. Data collection proceeded along two parallel but linked tracks. The first, ground-based track began with field plot layout and destructive sampling of 12 reed plots, followed by laboratory analysis of the harvested material to obtain dry weight and total organic carbon (TOC) content; these measurements were used to derive plot-level aboveground biomass (AGB) and carbon stock (Table 2) and to fit allometric regression models relating AGB to simple biometric predictors such as height, tiller density, and fresh weight. The second, remote-sensing track began with acquisition and pre-processing of Pléiades Neo satellite imagery of the study site, followed by Support Vector Machine (SVM) classification to delineate reed cover and extraction of vegetation indices (VIs) from the classified imagery. The two tracks were then brought together in a final step that correlated the satellite-derived vegetation indices against the field-derived AGB and carbon-stock values, yielding the remote-sensing regression models (Model-1 and Model-2) evaluated in Section 2.3 and Table 7.

### 4.1. Experimental Design

**Figure 21:**
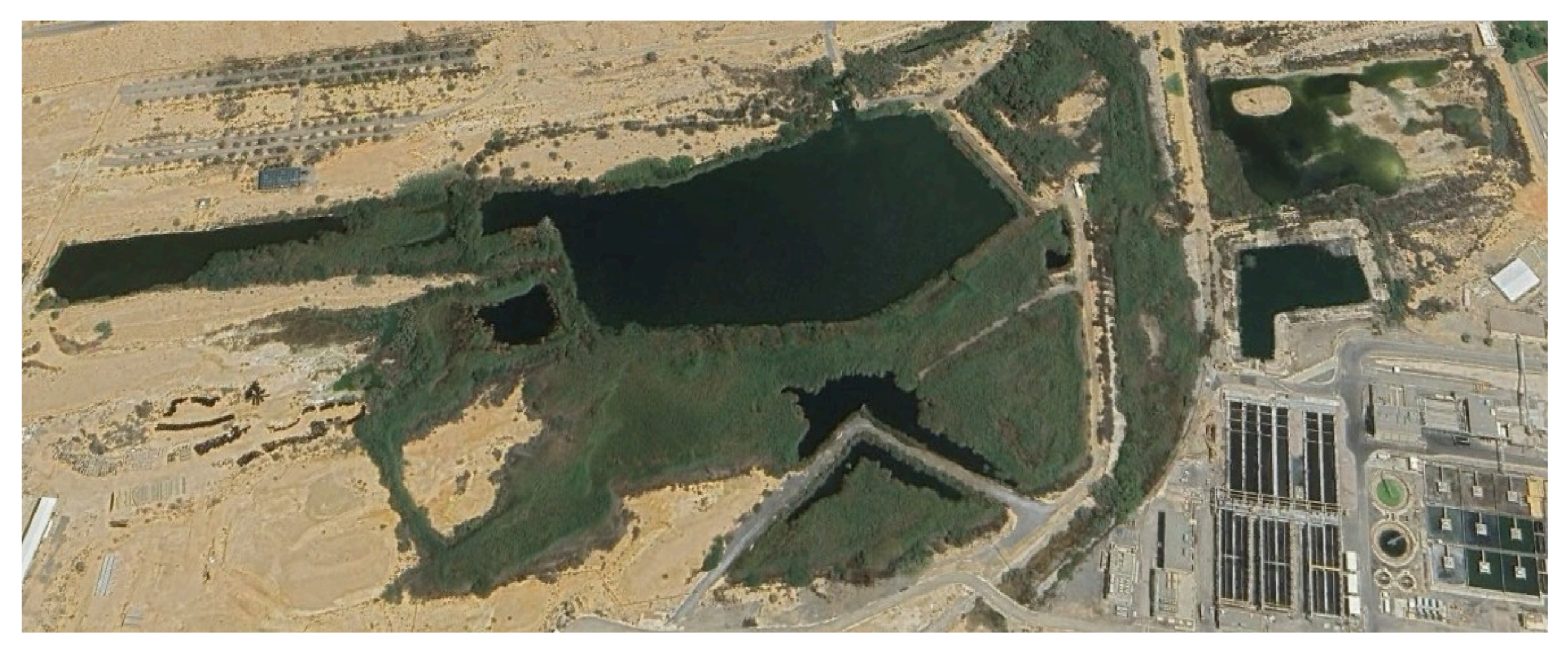
*Phragmites australis* study area.

**Figure 22:**
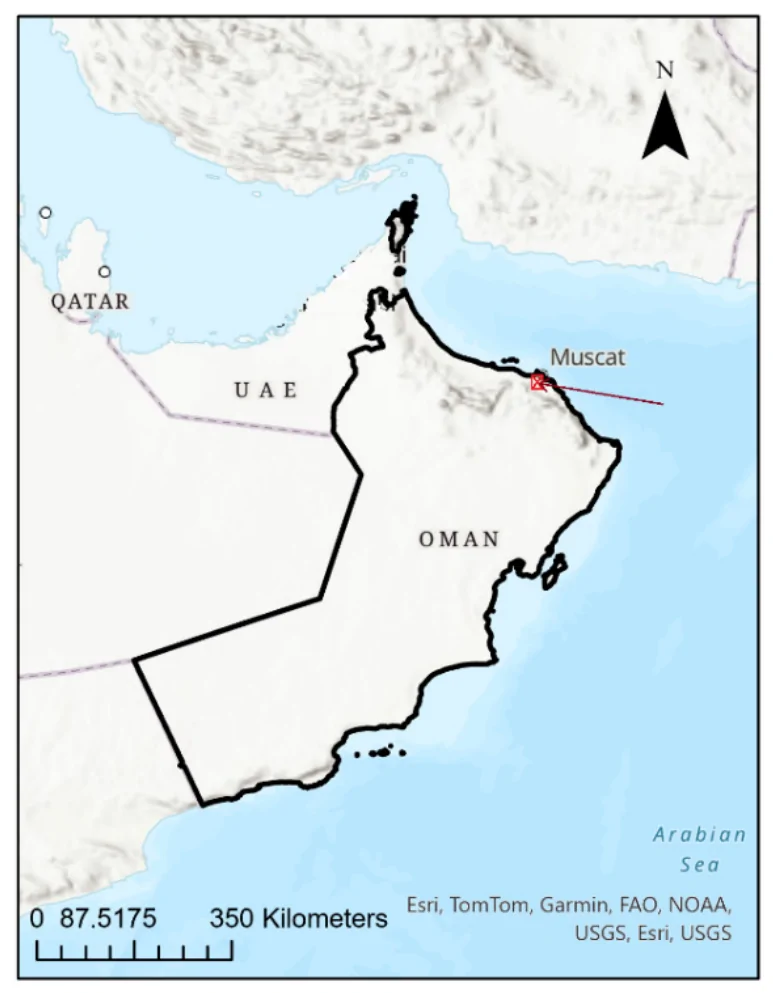
The location site (red square) of *Phragmites australis* study area in Oman.

The study of *Phragmites australis* (reedgrass) wetland was carried out in Al Ansab wetland located 15 km from the heart of Muscat, the capital of the Sultanate of Oman, at the precise coordinates 23°33′ 46.4076″ N, 58°20′1.86″ E (Figure 21 and Figure 22). Originally constructed on a smaller scale, the site was expanded towards the north-east by the addition of a buffer zone on 7 July 2008. Nowadays, the wetland encompasses a total land area of approximately 40 hectares (ha), which includes a lagoon and a range of associated habitats. As a man-made wetland, it is distinct from natural wetlands but still serves an important ecological function in providing a habitat for a variety of flora and fauna. The wetland processes and treats sewage effluent, resulting in clear water. The wetland is therefore a crucial site for ecological research and environmental conservation efforts in the region. A stratified random sampling approach was utilized to assess the aboveground biomass of *Phragmites australis*. This methodology falls under the category of destructive techniques and is aligned with the Clean Development Mechanism (CDM) methodologies overseen by the United Nations Framework Convention on Climate Change (UNFCCC).

The research site was subjected to thorough surveys using a random approach to gather data from plants for destructive sampling purposes. A total of 12 plots of *Phragmites australis*, measuring 1 m by 1 m, were sampled. The collected data included the number of reeds per plot, as well as their height, fresh weight, and coordinates. The precise locations of each plot were determined using a Global Positioning System (GPS) in combination with local mapping stations, guaranteeing an accuracy level of 0.1 m. This was done to mark the position of the sampling plot within the reed wetlands, ensuring accurate positioning of the randomly selected plots.

### 4.2. Sample Processing and Laboratory Measurements

**Figure 23:**
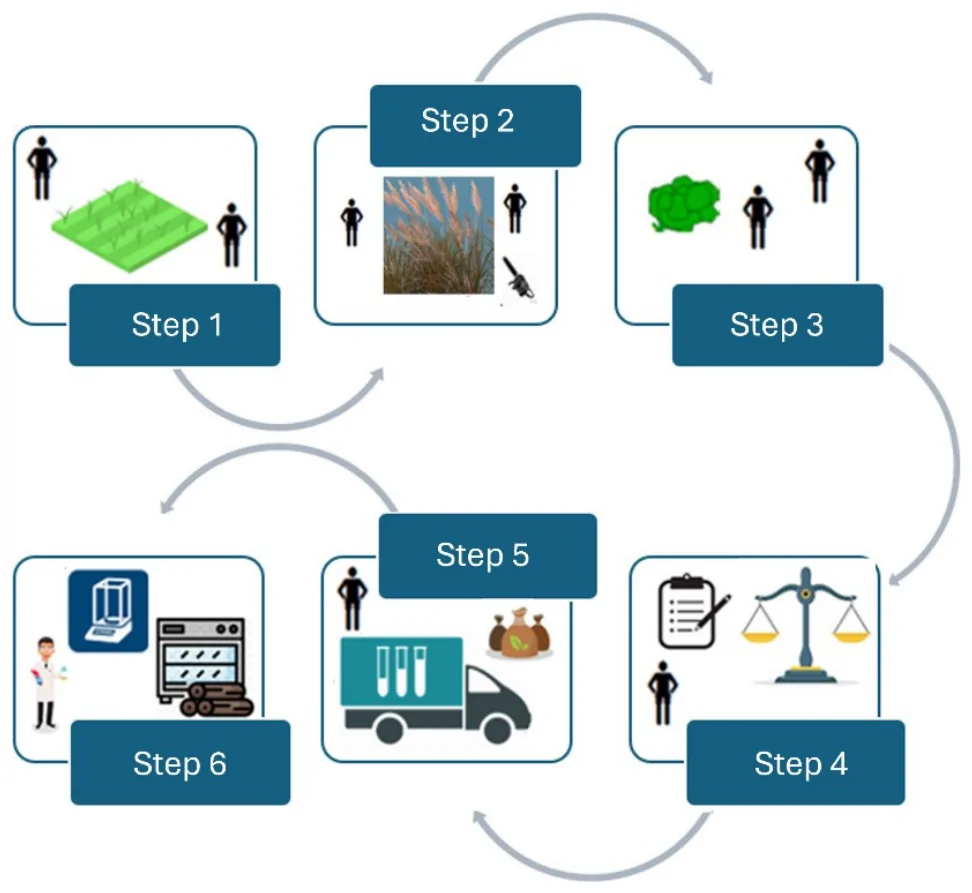
Field measurements and operational procedures depicted.

Figure 23 delineates the systematic procedure of the destructive sampling technique for the designated plots. The destructive sampling commenced with preliminary measurements in step 1. The diagram navigates through subsequent stages until step 6, where freshly gathered reed samples are transported to the laboratory for desiccation. The sequence initiates with the identification and measurement of reed plots for destructive sampling, followed by the implementation of the destructive procedure. The total weight of fresh reeds is then ascertained using a caliper, and subsamples are weighed, labeled, and dispatched to the laboratory for desiccation. Additionally, the height of the reeds and the number of stands per plot are measured. This visual representation offers a structured overview of the sampling process, ensuring precise data collection for a thorough analysis of carbon sequestration and associated variables.

The field data collection and measurements were carried out in three distinct phases.

Measurement of key biometric variables in standing reeds: This involved measuring the total height (m) and number of reeds per 1 m^2^.

Determination of fresh biomass: The stems of *Phragmites australis* were severed at ground level and subsequently tallied. After harvesting the 12 plots of reeds, the fresh aboveground biomass weight was measured.

Collection of samples for dry biomass determination: two samples of the mixture of each reed plot (ranging from 200 to 500 g) were collected to estimate its carbon content and dry process. These samples were prepared directly on site and passed to the university lab on the same day to be dried in an oven at 60–80 °C until a constant weight was achieved [14]. The samples were stored in sealed plastic bags and weighed on the same day of cutting using a Pitney Bowes scale (capacity: 0–2.5 kg). The 2nd sampling was carried out via the total organic carbon content (TOC) method to estimate the biomass by determining the amount of organic carbon present in the plant material. Carbon compounds were first extracted from 0.5 to 1 g of each reed’s dried plot sample using a mixed-acid digestion method (0.0125 M H2SO4 and 0.05 M HCl); the resulting digested extract was then analyzed for organic carbon content using a TOC-V analyzer via elemental analysis/combustion.

The procedure for assessing dry biomass adhered to established protocols detailed in the SOP wood density [50] and SOP destructive sampling [35]. However, this study employed two distinct methods to estimate the carbon sequestration of *Phragmites australis* (reedgrass): one involved dry sampling biomass steps, while the other utilized the laboratory total organic carbon concentration method. In this context, reeds were classified as non-woody plants for these assessments.

A total of 12 plots containing *Phragmites australis* specimens were evaluated, with samples meticulously stored in plastic bags to ensure their integrity and prevent moisture loss. These samples were subsequently transported to the Sultan Qaboos University (SQU) laboratory for further analysis using both methods. To estimate the biomass of the reed plots, full-weight calculations and subsamples were utilized. Each of the 12 plot samples was taken to the lab’s drying room, where they were dried and weighed to determine the dry weight of the entire plots. This methodology, in accordance with IPCC Methodological Guidelines [9], involved the systematic randomization of sampling plots for *Phragmites australis* across accessible zones. This randomization process utilized GPS coordinates obtained with an LT700H 8″ Android RTK Tablet and GNSS Antenna AT312 manufactured by CHCNAV (Shanghai Huace Navigation Technology Ltd.), Shanghai, China.

Upon arrival at the laboratory, samples were weighed to determine fresh weight. Following initial weighing, samples underwent air-drying in paper bags to remove excess moisture before being oven-dried at 60–80 °C, following [35,36,37] and IPCC, 2014 methodology [9]. Daily weighing during drying determined stable dry weight, allowing calculation of the percentage of dry weight relative to original fresh weight using the dry-to-fresh factor (DF), facilitating the determination of the ratio between dry weight and original fresh weight for each sample.

*Dry to wet ratio* = *SDW*/*SWW * (1)

*Dry weight* (kg) = *ADWR*∙*FPW* (2)

where SDW is the sample dry weight (kg) and SWW is the sample wet weight (kg).

Furthermore, the equation described in [36], which was originally created for non-woody plants, could be used to calculate the dry biomass of the reeds:

*ODW* = *TFW* × (*SODW*/*SFW*) (3)

where ADWR is the average dry-to-wet ratio, FPW is the full plot weight (kg), ODW is the total oven dry weight, TFW is the total fresh weight (kg), SFW = sample fresh weight (kg), and SODW = sample oven dry weight (kg).

On the other hand, the total organic carbon (TOC) method estimates biomass by determining the amount of organic carbon present in the plant material. This method involves collecting plant samples, drying them to remove moisture, and then analyzing the carbon content using laboratory techniques such as elemental analysis or combustion. The concentrations of total organic carbon (TOC) in the reedgrass samples were quantified using a TOC analyzer, a widely recognized and reliable instrument for determining carbon content. The TOC method provides an indirect estimation of biomass based on the assumption that a certain proportion of the plant’s organic carbon is derived from biomass. The actual proportion can vary depending on the plant species and environmental factors, so it is essential to calibrate the method for specific ecosystems.

The carbon stock in the reedgrass samples was subsequently calculated based on the TOC concentration, expressed as a percentage, multiplied by the corresponding dry weight of the sample. The resulting carbon stock values were then expressed in grams per square meter (g/m^2^), providing a quantitative measure of the carbon content within the reedgrass ecosystem. By employing this rigorous methodology, encompassing sample collection, preparation, analysis, and calculation, a comprehensive evaluation of the carbon stock in the reedgrass samples was accomplished, allowing for a more profound understanding of the carbon dynamics within the studied ecosystem.

The allometric relationship between plant height and aboveground biomass (AGB) was modeled using a power function of the following form:

*AGB = a × H^b^* (4)

where AGB represents aboveground biomass (kg/m^2^), H is plant height (m), and *a* and *b* are regression coefficients determined empirically.

In addition, carbon stock (C) was estimated using:

*C = AGB × CF* (5)

where CF represents the carbon fraction derived either from dry biomass or measured total organic carbon (TOC) content.

### 4.3. Aboveground Biomass and Carbon Sequestration

A specified number of individual plots of *Phragmites australis* (reedgrass) were selected from the targeted area to measure various parameters such as reed height (H), the number of reed stems per plot, and the total fresh weight per plot. Reed height measurements were obtained using an Electronic Clinometer. Subsequently, all selected reed plots were harvested to determine their respective fresh weights, which constituted the total aboveground biomass (AGB). In the field, fresh samples were weighed, mixed well, and subsamples were placed in marked bags. The subsamples were then utilized to establish an oven dry-to-wet mass ratio, which was subsequently used to convert the total wet mass to oven dry mass. A subsample was taken to the laboratory and oven-dried until a constant weight was achieved in accordance with the methods outlined by [36,37].

The initial approach involved the use of the dry-to-fresh ratio method, which calculates biomass by considering the weight ratio of dried reed samples to their corresponding fresh weight. The second method, known as the total organic carbon (TOC) method, estimates aboveground biomass (AGB) by measuring the total organic carbon content of reed samples. Alongside these methods, insights from [48], a study on similar plant species, were incorporated. Conti’s research [48] provided a valuable benchmark for evaluating the performance of the dry-to-fresh ratio and TOC methods when applied to reeds.

Externally derived allometric equations were tested to estimate AGB from field-measured predictors, where AGB represents the total aboveground biomass of individual reed plots measured in 1 m × 1 m and expressed in kilograms (kg). CD refers to the canopy diameter of the plot in meters (m), and H represents the height of the reeds in meters (m), with “e” symbolizing the base of the natural logarithm. Four distinct externally derived models, each using a single predictor (height, canopy diameter, or stem count), were sourced from relevant studies and compared based on their correlation indicators (R2 values) against the actual measured destructive aboveground biomass; Equation 9 shows the diameter-based model as a representative example, with H omitted from this particular equation because it is a single-predictor (diameter-only) model.

H: height of reeds (m), and D: plot canopy diameter CD (m).

Through a meticulous examination of both the dry-to-fresh ratio and TOC methods, along with insights gleaned from Conti et al. [48] study, the researchers sought to gain a comprehensive understanding of the most effective and accurate approach for estimating the aboveground biomass of reeds. By comparing these methodologies, this study aimed to contribute valuable insights to the field of wetland vegetation biomass estimation and advance our knowledge of ecological modeling.

The aboveground reed biomass was calculated by evaluating the total weights of the reeds above the harvested ground. It is worth noting that plant tissues are commonly cited in the literature as containing between 45 and 50% carbon, a figure applied to both dead and live samples [35]; however, as detailed below, this range is derived primarily from woody tissue and does not directly transfer to herbaceous species such as *Phragmites australis*. At a global scale, carbon contents in plant organs vary, ranging from 45.0% in reproductive organs to 47.9% in stems, particularly in the woody parts [51]. Although there is no universally accepted conversion value for biomass to carbon content, the IPCC 2006 guidelines [52] suggest a range of 45% to 50%, which is commonly adopted as the default value for many trees. The assessment of the carbon storage capacity of plants involved integrating biomass values with a carbon default value or carbon factor (CF) typically below 0.50 [53,54]. To convert the carbon content into CO2 equivalent (CO2eq), the carbon quantity was multiplied by 3.67, reflecting the ratio of the molecular weight of carbon dioxide to the atomic weight of carbon [55].

It should be noted that the 45–50% range cited above, and the 0.50 default factor applied in this study’s dry-weight-ratio approach, are derived primarily from woody plant tissue [35,53,54]. A more species-specific benchmark is available from direct carbon-content measurements of *Phragmites australis* itself: Silan et al. [38] report a carbon fraction of 0.43–0.45 (mean ≈ 0.44) for this species, consistent with the 0.40–0.45 range reported by Verma et al. [39] across 117 herbaceous species more broadly. We therefore also report a third, intermediate carbon-stock estimate using this 0.44 species-specific factor alongside the 0.50 IPCC-default and TOC-based estimates (Table 2), to allow direct comparison with this more targeted literature value. Direct TOC analysis of this study’s own reed samples measured a substantially lower organic carbon fraction (10.8–25.2%, mean 17.3%)—expressed, as throughout this study, on a dry-weight basis (g organic carbon per g oven-dried sample mass, not fresh mass); we have re-checked this calculation and confirm it is not a fresh-weight artefact. Even the species-specific 0.44 literature value remains far above this measured fraction, indicating that woody-tissue carbon defaults—and, to a lesser extent, even herbaceous-tissue defaults from other species—likely overestimate carbon content in *Phragmites australis* at this site, or that the TOC measurement is itself depressed by a non-carbon component of the sampled dry matter; this discrepancy and its possible sources are examined further in the Discussion.

The determination of total carbon storage in vegetation involves the multiplication of vegetation biomass, the carbon conversion factor, and the area under consideration [42]. The carbon conversion factor denotes the proportion of carbon content within the vegetation and exhibits variability across different vegetation types [42]. For the purpose of this study, the carbon storage assessment focused exclusively on the predominant vegetation of the estuary salt marsh communities, namely *Phragmites australis*, which possesses a carbon conversion factor of approximately 0.50, consistent with the value applied throughout this study. It is important to note that this specific value applies solely to *Phragmites australis*. The estimation of carbon storage values was conducted as follows:

*Cplant* = *SB*∙0.50∙*Aplant* (6)

where Cplant is the carbon storage in the Phragmites community (g), SB is the aboveground standing biomass of Phragmites (g/m^2^), and Aplant is the area occupied by the Phragmites community (m^2^).

Table 2 summarizes the carbon stock measurements conducted on *Phragmites australis* plants. Across the 12 sampled plots in this study, the dry-weight ratio approach yielded an average AGB of 2.22 kg/m^2^ and a corresponding average carbon stock of 1.11 kg/m^2^ (applying the 0.50 carbon conversion factor, Table 2). The TOC analyzer approach, which measures carbon content directly rather than deriving it from AGB, gave an average carbon stock of 0.38 kg/m^2^ (mean TOC content of 17.3% applied to dry AGB, Table 2). Reference [40] examined dry samples and reported an AGB of 1.853 kg/m^2^, with a carbon content of 0.81 kg/m^2^, specifically representing 80% of the belowground biomass (BGB) in the range of 1.46–3.3 kg/m^2^. Reference [41] investigated the carbon stock in pristine salt marsh and found a range of 0.642 kg/m^2^ to 3.0–35.6 kg/m^2^. Additionally, impacted salt marshes exhibited carbon stock densities ranging from 1.04 kg/m^2^ to 8.6–24.0 kg/m^2^. Reference [43] observed a wide range of carbon stock densities, varying from 0.31 to 52.90 Mg/ha (mean: 0.733 kg/m^2^).

Reported aboveground biomass (AGB) and carbon content measurements exhibit significant variation across different methods and locations, ranging from 1.6 to 62.3 kg/m^2^. In this study, the average AGB was estimated at 2.22 kg/m^2^ using the dry-weight ratio approach, with a corresponding average carbon stock of 1.11 kg/m^2^ (dry-ratio) and 0.38 kg/m^2^ (TOC approach). Comparatively, other studies reported AGB values such as 1.853 kg/m^2^ from dry samples, 0.642 kg/m^2^ in pristine salt marshes, and 1.04 kg/m^2^ in impacted salt marshes. Larger-scale estimates include Egypt with 30.4 Mg ha^−1^ year^−1^ (3.04 kg/m^2^), Iraq with 13.6 Mg ha^−1^ year^−1^ (1.36 kg/m^2^), Minnesota with 16.8 Mg ha^−1^ year^−1^ (1.68 kg/m^2^), and Sweden with values ranging from 9.9 to 25.1 Mg ha^−1^ year^−1^ (0.99–2.51 kg/m^2^). These variations highlight the influence of environmental conditions and methodological differences on biomass and carbon stock estimations.

Overall, the table highlights the variability in carbon stock measurements in *Phragmites australis* plants across different locations and methodologies. These findings contribute to our understanding of the carbon dynamics in this plant species and its implications for carbon sequestration and ecosystem carbon storage.

Reed carbon stocks storage (CSS), in kg, was appraised through the conversion of the computed destructive biomass of the reed plots, employing a standard factor of 0.50 as applied throughout this study, assuming the reeds as non-woody plants [42]. Moreover, for determining the CO2 equivalent (CO2eq), the amount of carbon was multiplied by 3.67, representing the ratio between the molecular weight of carbon dioxide and the atomic weight of carbon [55].

*CSS* = *TDR*∙0.50 (7)

where CSS is the carbon stock storage (kg), and TDR is the reed dry biomass (kg).

*CO*2_*eq* = *CSS*∙3.67∙0.001 (8)

where 0.001 = the factor for converting CO2eq from kg to tons.

### 4.4. Sampling and Analytical Method—Statistical Analyses

The process employed to construct allometric models for aboveground biomass (AGB) involved regression analysis, where the aboveground biomass was regressed against independent variables such as reed height (H), canopy diameter (CD), and the number of reed stems per plot, with Ln (AGB) serving as the dependent variable. Two approaches for calculating AGB were considered: one derived from dry biomass (dry-ratio AGB) and the other from total organic carbon (TOCAGB), both correlated as dependent variables with the independent variables of the reed plots.

Initially, the correlation between the actual AGB obtained through destructive methods and the tested generalized allometric equations, outlined in Equation (9) [48], was examined using Minitab 19.0 software for data analysis. Separate log–log models were fitted for each individual allometric variable to identify the most effective predictor for AGB. The evaluation of these log–log models incorporated crucial metrics such as R2 and *p*-Value.

*AGB* = *e*^((−0.370 + 1.903(ln(*D*))) (9)

where H is the height of reeds (m) and D is the plot canopy diameter (CD) (m).

Statistical analyses were conducted to evaluate the relationships between measured plant variables and biomass estimates. Regression models (linear and nonlinear) were developed to assess the predictive performance of selected variables, particularly plant height, in estimating aboveground biomass (AGB). Model performance was evaluated using standard statistical indicators, including the coefficient of determination (R^2^), mean absolute error (MAE), and root mean square error (RMSE). This assessment focused exclusively on the fitting dataset to assess the performance of local models and compare them against previously established models. The biomass of each reed was then calculated using the previously tested equations and the most suitable equation developed in this study. A comparison was carried out using an ANOVA test.

### 4.5. Testing Established Allometric Model Equation

In this investigation, the biomass estimated at the plot scale using the allometric equation was referred to as the observed biomass. The study conducted by [48] served as the fundamental basis for our research in formulating the allometric equations, along with relevant studies focusing on similar plant species. Conti’s study extensively analyzed and assessed prior research concerning actual destructive plants, accounting for errors and limitations. Their protocol was recommended for applying general aboveground biomass (AGB) models and involved the development of allometric equations for predicting individual aboveground biomass.

Consequently, datasets from destructively sampled reed biomass were compared to assessing their correlation indicators, specifically R2 values, with the tested equations in area-based biomass assessment. The assessment of *Phragmites australis* (reedgrass) involved 12 destructive plots, showcasing linear relationships with high R2 values for most of the assessed equations.

### 4.6. Developing a Local Remote Sensing Model

The estimation of aboveground biomass (AGB) in the *Phragmites australis* (reedgrass) area of interest was conducted using both the destructive method and the TOC method along with remote sensing method. Pléiades Neo satellite images were used in this approach; the images were obtained prior to the destructive sampling of plants. The acquisition date for these images was 28 August 2023, and the image had a panchromatic resolution of 30 cm with seven spectral bands: deep blue, blue, green, red, red edge, near-infrared, and panchromatic. The red-edge band is required for the red-edge-dependent vegetation indices (NDRE, RE_SER) used in this study; standard Pléiades (1A/1B) imagery, which lacks a red-edge or deep-blue band, would not support these indices. High-resolution ortho mosaics were used to compute various vegetation indices (VIs), and corresponding raster layers were created based on these indices.

To minimize interference stemming from soil, roads, water bodies, and weeds within the raster layers, binary masks were created using orthorectified images and the Support Vector Machine (SVM) as a classifier. Zonal statistics techniques were subsequently employed to ascertain the average pixel values at each sampling location across these raster layers, establishing correlations with ground-based biomass measurements. To streamline and standardize these procedures, ArcGIS Desktop 10.7 (Esri, Redlands, CA, USA) and a machine learning-driven programming language were utilized to automate the extraction of vegetation indices (VIs) and the removal of soil/weed interference.

Various vegetation indices (VIs) were computed using data from near-infrared to red (NIR-R), near-infrared to red-edge (NIR-RE), and Spectrometer sources. The recommended VIs (outlined in Table 8) in this study were formulated using digital numbers (DNs) derived from red, green, and blue bands (RGB) within the captured images. These proposed VIs were employed to evaluate band characteristics related to biophysical properties such as leaf greenness and chlorophyll content. Additionally, adjustments were made to the inherent factors of specific VIs, such as the correction factor in the soil-adjusted vegetation index (SAVI).

Remote sensing data were integrated to evaluate the potential for large-scale biomass estimation. Spectral information was used to calculate vegetation indices, including normalized difference vegetation index (NDVI), soil-adjusted vegetation index (SAVI), and near-infrared-based indices (e.g., NIR-R and NIR-RE). These indices were correlated with field-measured biomass data to assess their effectiveness in predicting spatial variability in aboveground biomass. This approach enables extrapolation of plot-level measurements to broader spatial scales. A sequence of commonly utilized vegetation indices (VIs) was computed using high-resolution ortho mosaics, generating corresponding raster layers (Table 8). Binary masks were then created based on orthorectified images to remove interference from soil, roads, water, and weeds, employing Support Vector Machine (SVM) as a classifier. Zonal statistics were subsequently applied to determine average pixel values across these layers at each sampling location, establishing associations with ground-based biomass measurements.

To streamline and standardize the processes, the ArcGIS library and programming language were utilized for automating VI extraction and eliminating soil/weed interference. The process of plant delineation through spectral analysis indices and composite layers commenced with investigating all single and combined vegetation indices, selecting the most suitable indices based on available satellite image sensor bands (R, G, B, near infrared, red edge, and deep blue). Following this, indices were stacked to construct a composite layer using the composite command from ArcGIS software modeling. A supervised classification was then conducted on the composite images using the support vector machine method, incorporating field measurements to delineate targeted plants in the classification process.

Detailed classifier parameters (kernel type) and formal accuracy metrics (overall accuracy, Kappa coefficient) for this classification step were not recorded during the original analysis. This is a limitation of the remote-sensing workflow reported here; future work on this system should incorporate a formal accuracy assessment (e.g., a confusion matrix with held-out validation samples) to support reproducibility. To be explicit about the scope of validation in this study, the biomass-prediction models built from the classified vegetation indices were cross-validated against destructively measured AGB (Section 2.3), but the upstream SVM vegetation classification itself was not independently accuracy-assessed; the reported biomass cross-validation results should therefore not be read as validating the classification step, which remains a distinct and unverified source of potential error.

Additionally, a classification map was generated to depict various classes such as soil, roads, buildings, crops, and delineated plants in separate categories. Subsequently, the class representing delineated plants was selected from the classification map and exported as a feature class, which would be used for AGB estimation correlation modeling.

Remote-sensing techniques were utilized for data collection regarding plant biomass and canopy area index, serving as input for machine learning algorithms dedicated to estimating the carbon sequestration capacity of these plants. Concurrently, Geographic Information Systems (GISs) played a pivotal role in integrating spatial data, enabling the creation of precise maps delineating the study area.

**Figure 24:**
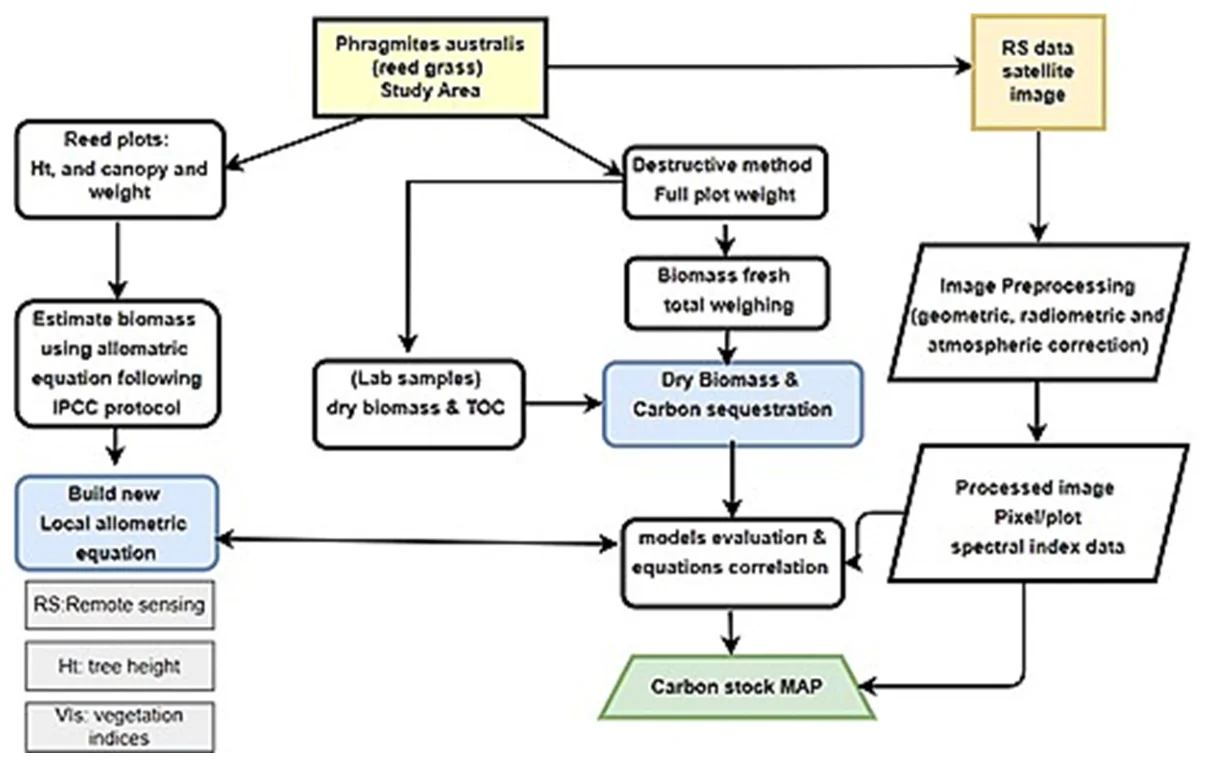
Summary of the approach and analysis steps followed in this study.

The methodology and analysis steps undertaken in this research are illustrated in Figure 24, showcasing a process involving a destructive method. This method entails gathering plant material to acquire essential data for estimating the carbon stock within the plants. The destructive approach involves collecting samples of plant tissue for subsequent laboratory analysis, including techniques like carbon analysis. The data obtained through the destructive method were then compared and correlated with information gathered through remote sensing and GIS.

## 5. Conclusions

In hyper-arid artificial wetlands, accurate assessment of ecosystem services such as carbon sequestration requires methods adapted to stress-driven plant variability. While *Phragmites australis* maintains measurable aboveground carbon stocks at the Al Ansab site, conventional allometric models fail under conditions of morphological plasticity, and field-measured height alone correlated only weakly with destructively measured biomass in this dataset. Applying the same rigorous, plot-level cross-validation standard to the satellite vegetation-index models (Section 2.3) produced a consistent result across both approaches, establishing the specific sampling scale that operational biomass prediction at Al Ansab will require. This study is limited to aboveground biomass; belowground rhizome and root carbon, which can represent a substantial share of total carbon storage in Phragmites stands, was not quantified and should be included in future carbon-budget assessments of this system. The findings are based on a single 40-hectare constructed wetland sampled at one time point (n = 12 plots for destructive sampling; eight plots with satellite coverage), and the remote-sensing classification accuracy could not be formally verified; the natural next step is a substantially larger, multi-season dataset with broader satellite coverage, applying the same validation standard established here to confirm operational carbon-monitoring tools for hyper-arid constructed wetlands, in this and other Gulf Cooperation Council sites. This study’s principal contribution is therefore twofold: a corrected, transparent quantification of destructive sampling-based carbon stocks at this site (Table 2), and a rigorous, quantitative benchmark for the sampling design and cross-validation standard that future predictive modeling in this system will need to meet.

## Figures and Tables

**Table 1:** List of actual destructive *Phragmites australis* (reedgrass) plot measurement results. Ht: reed height; Et: weight.

Plot No	Plot Size (m)	Reeds Height (m)	Reeds per Plot (No)	Reeds Plot Fresh Weight (kg/m^2^)	Sample Dry Weight (g)
1	1 × 1	3.15	214	3.7	430
2	1 ×1	2.8	138	2.8	390
3	1 × 1	3	150	2.2	390
4	1 × 1	2.5	180	3.5	380
5	1 × 1	3.6	45	4.4	450
6	1 × 1	3.2	53	6.5	340
7	1 × 1	3.6	51	6.1	360
8	1 × 1	2.6	60	4.3	370
9	1 × 1	3.91	73	7.5	480
10	1 × 1	4.3	62	4.9	420
11	1 × 1	2.2	115	4.1	500
12	1 × 1	3.92	72	4.9	490

**Table 2:** List of actual destructive *Phragmites australis* (reedgrass) plot measurements using a two-way approach. Ht: reed height; Et: weight.

		Dry Weight Ratio Approach		TOC Approach	
Plot No	Reed Wet–Dry Ratio	Reed AGB kg/m^2^ from Dry Ratio	Reed Carbon Stock kg/m^2^ from Dry Ratio	TOC %	Carbon Stock kg/m^2^ from TOC Analyzer
1	0.488	1.81	0.90	17.9	0.3240
2	0.538	1.51	0.75	12.8	0.1933
3	0.744	1.64	0.82	20.2	0.3313
4	0.526	1.84	0.92	10.8	0.1987
5	0.467	2.05	1.03	16.8	0.3444
6	0.500	3.25	1.63	12.3	0.3997
7	0.306	1.86	0.93	16.1	0.2995
8	0.432	1.86	0.93	25.2	0.4687
9	0.521	3.91	1.95	17.5	0.6843
10	0.452	2.22	1.11	17.5	0.3885
11	0.560	2.30	1.15	20.2	0.4646
12	0.490	2.40	1.20	20.3	0.4872
Average Carbon stock:	1.11			0.3820	

Note: applying the species-specific carbon factor of 0.44 reported for *Phragmites australis* by Silan et al. [38] (and consistent with the 0.40–0.45 range for herbaceous species reported by Verma et al. [39]), rather than the generic IPCC 0.50 default, gives an intermediate average carbon stock of 0.98 kg/m^2^ for the dry-weight-ratio approach.

**Table 3:** Comparison of aboveground carbon stock and biomass estimates for *Phragmites australis* and comparable wetland vegetation from this study and the published literature. The first six rows report standing carbon/biomass stocks (kg/m^2^); the last four rows (Egypt, Iraq, Minnesota, Sweden) report annual productivity rates (Mg ha^−1^ yr^−1^, converted to kg/m^2^ yr^−1^ for reference) and are not directly comparable to the standing-stock rows above. The “Comments” column reports supplementary figures as stated in each cited source (e.g., a reported range, or a belowground-biomass estimate distinct from the AGB column); the “AGB” column consistently reports the single aboveground biomass or carbon-stock value used for comparison in the text.

AGB	Comments	Method/Location	Reference
2.22 kg/m^2^ AGB	1.11 kg C/m^2^ *	Dry/fresh ratio approach	This Study
	0.38 kg C/m^2^	TOC approach	
1.853 kg/m^2^	1.46–3.3 kg/m^2^ (80% BGB estimate)	Dry samples	[40]
0.642 kg/m^2^	Range 3.0–35.6 kg/m^2^	Pristine Salt Marsh	[41]
1.04 kg/m^2^	Range 8.6–24.0 kg/m^2^	Impacted Salt Marsh	
0.31–52.90 Mg/ha (mean: 0.733 kg/m^2^)			[42,43]
30.4 (Mg ha^−1^ Year^−1^) 3.04 kg/m^2^		Egypt	[44]
13.6 (Mg ha^−1^ Year^−1^) 1.36 kg/m^2^		Iraq	[45]
16.8 (Mg ha^−1^ Year^−1^) 1.68 kg/m^2^		Minnesota	[46]
9.9–25.1 (Mg ha^−1^ Year^−1^) 0.99–2.51 kg/m^2^		Sweden	[47]

* A 0.50 carbon factor is used; applying the species-specific factor of 0.44 reported for *Phragmites australis* by Silan et al. [38] (consistent with Verma et al. [39]) instead gives 0.98 kg C/m^2^ (see the note below Table 2 and the Discussion). Note: Values in this table are reported in the original units used by each cited source. Conversion factors applied for comparison in the text: 1 Mg/ha = 0.1 kg/m^2^; 1 Mg ha^−1^ year^−1^ (dry biomass, oven-dry basis) = 0.1 kg/m^2^ year^−1^.

**Table 4:** The list of *Phragmites australis* (reedgrass) AGB logarithms-driven regression models using the measured plant plots (n = 12).

Plots (12) Model#1 Stepwise	Regression Equation Model AGB (kg/m^2^) = −0.03139 + 0.6520 LN Height (m)	Variables H	R-sq (pred) 100.0%	*p*-Value Fit
Model#2 Stepwise	AGB = −0.4534 + 0.6520 LN Height (m)	H	100.0%	Fit
LN Dry	AGB = −0.90 + 0.030 LN Height (m) + 0.756 LNF weight (kg)	H&W	24.07%	0.022:0.9280.10&0.4
Regression (AGB) Stepwise LN TOC	+ 0.114 LN (grass/plot) −0.183+ 0.642 LNF weight (kg) AGB = −0.78 + 0.038 LN Height (m) + 0.547 LNF weight (kg)	W H&W	40.21% 0.00%	0.001:0.001 0.275:0.951,0.226,
Regression (AGB) Stepwise	−0.064 LN (grass/plot) − 1.141 + 0.626 LNF weight (kg)	RN and W	0.00%	0.039:0.039

**Table 5:** The list of novel models of three targeted species on the estimations of aboveground biomass using statistical correlation regression modeling analysis.

Species	Model (Ln (AGB))	Variable	Model No
*Phragmites australis*	AGB = −0.03139 + 0.6520 LNHeight (m) AGB = −0.4534 + 0.6520 LNHeight (m)	H H	1 2

**Table 6:** Models’ performance fitness with regard to the destructive sampling (dry biomass and TOC methods). The MAE and RMSE are reported in kg/m^2^ (AGB for the dry-biomass row; carbon stock for the TOC row); the MAPE is computed on back-transformed (real-space) predictions. The TOC row uses the corrected carbon-stock values (Section 2.1).

Models’ Fitness to Destructive Dry Biomass	R2	MAE	RMSE	RE	MAPE
Model-1	0.100	0.450	0.636	−3.8%	18.0%
Model-2	−1.518	0.861	1.064	−46.1%	35.1%
TOC					
Model-1	−25.99	0.655	0.672	40.2%	200.8%
Model-2	−5.30	0.298	0.324	−2.1%	97.3%

**Table 7:** RS versus predicted models’ fitness performance. The MAE and RMSE are reported in kg/m^2^ (AGB); the MAPE is computed on back-transformed (real-space) AGB predictions. The R^2^ shown reflects fit to a height-derived AGB proxy (Model#1/Model#2, Section 2.2), not to destructively measured AGB directly; see the Section 2 for a separate, plot-level cross-validated assessment against measured AGB, which found no combination of the tested predictors to be a validated predictor of biomass at this sample size.

Models’ Fitness	R2	MAE	RMSE	RE	MAPE
Model-1	0.781	0.111	0.134	0.20%	5.38%
Model-2	0.783	0.073	0.088	0.20%	5.38%

**Table 8:** A list of some vegetation indices extracted and tested in the study.

Vegetation Spectral Indices	Formula Definition	References
Red (R), green (G), blue (B)	Raw value of each band rn = R/(R + G + B);	/
Normalized red (rn), green (gn), blue (bn)	rn = R/(R + G + B); gn = G/(R + G + B); bn = B/(R + G + B)	/
* Green/red ratio index (GRRI)	GRRI = G/R	/
* Green/blue ratio index	GBRI = G/B	/
* Red/blue ratio index (RBRI)	RBRI = R/B	/
* Color intensity index (CII)	INT = (R + G + B)/3	[56]
Green leaf index	GLI = (2 × G − R − B)/(2 × G + R + B)	[57]
* Visible atmospherically resistance index (VARI)	VARI = (G − R)/(G + R − B)	[58]
Vegetative index	VEG = G/(Ra × B(1 − a)), a = 0.667	[59]
* Normalized difference red edge (NDRE)	NIR-RE/NIR + RE	[60]
* Normalized difference vegetation index (NDVI)	NIR-R/NIR + R	[61]
* Simple ratio vegetation index (SRVI)	NIR/R	[62]
* Canopy chlorophyll content index (CCCI)	NDRE/NDVI	[60]
SAVIcam (noise-adjusted vegetation index)	(1.5N)(B − R)/(B + R + 0.5N)	[63]
* Transformed soil-adjusted vegetation index	(s * (NIR − s * Red − a))/(a * NIR + Red − a * s + X * (1 + s2))	[64]

Note: N = 255. while R, G, and B are mean of digital number (DN) of these respective bands. RE1 RED edge band 1—* The tested index with good respond.

## Data Availability

The data presented in this study is available on request from the corresponding author.

## Abbreviations

The following abbreviations are used in this manuscript:

AGB	Aboveground Biomass	H	Reed Height
ADWR	Average Dry to Wet Ratio	RSAGB	Remote Sensing Aboveground Biomass
BGB	Belowground Biomass	RMSE	Root Mean Square Error
BAF	Bioaccumulation Factors	R^2^	Coefficient of Determination
CD	Canopy Diameter	SDW	Sample Dry Weight
C	Carbon	SODW	Sample Oven Dry Weight
CSS	Carbon Stock Storage	SWW	Sample Wet Weight
CDM	Clean Development Mechanism	SAVI	Soil-Adjusted Vegetation Index
GPS	Global Positioning System	SQU	Sultan Qaboos University
LiDAR	Light Detection and Ranging	SAR	Synthetic Aperture Radar
MAE	Mean Absolute Error	TFW	Total Fresh Weight
MAPE	Mean Absolute Percentage Error	TOC	Total Organic Carbon
Ln	Natural Logarithm	TOCAGB	Total Organic Carbon Aboveground Biomass
NIR-RE	Near-Infrared to Red-Edge	TF	Translocation Factor
NIR-R	NIR to Red Ratio	TDR	Total Reed Dry Biomass
ODW	Oven Dry Weight	VIs	Vegetation Indices
Q^2^	Cross-Validated (Leave-One-Out) Coefficient of Determination	CAGB	Calculated (Height-Derived) Aboveground Biomass
